# A polysaccharide utilization locus from *Flavobacterium johnsoniae* enables conversion of recalcitrant chitin

**DOI:** 10.1186/s13068-016-0674-z

**Published:** 2016-11-28

**Authors:** Johan Larsbrink, Yongtao Zhu, Sampada S. Kharade, Kurt J. Kwiatkowski, Vincent G. H. Eijsink, Nicole M. Koropatkin, Mark J. McBride, Phillip B. Pope

**Affiliations:** 1 Department of Chemistry, Biotechnology and Food Science, Norwegian University of Life Sciences (NMBU), 1432 Ås, Norway; 2 Wallenberg Wood Science Center, Division of Industrial Biotechnology, Department of Biology and Biological Engineering, Chalmers University of Technology, 412 96 Gothenburg, Sweden; 3 Department of Biological Sciences, University of Wisconsin—Milwaukee, Milwaukee, WI 53201 USA; 4 Department of Microbiology and Immunology, University of Michigan Medical School, Ann Arbor, MI 48109 USA

**Keywords:** Polysaccharide utilization locus, Chitin, Recalcitrant polysaccharides, Bacteroidetes

## Abstract

**Background:**

Chitin is the second most abundant polysaccharide on earth and as such a great target for bioconversion applications. The phylum Bacteroidetes is one of nature’s most ubiquitous bacterial lineages and is essential in the global carbon cycle with many members being highly efficient degraders of complex carbohydrates. However, despite their specialist reputation in carbohydrate conversion, mechanisms for degrading recalcitrant crystalline polysaccharides such as chitin and cellulose are hitherto unknown.

**Results:**

Here we describe a complete functional analysis of a novel polysaccharide utilization locus (PUL) in the soil Bacteroidete *Flavobacterium johnsoniae*, tailored for conversion of chitin. The *F. johnsoniae* chitin utilization locus (ChiUL) consists of eleven contiguous genes encoding carbohydrate capture and transport proteins, enzymes, and a two-component sensor–regulator system. The key chitinase (ChiA) encoded by ChiUL is atypical in terms of known Bacteroidetes-affiliated PUL mechanisms as it is not anchored to the outer cell membrane and consists of multiple catalytic domains. We demonstrate how the extraordinary hydrolytic efficiency of ChiA derives from synergy between its multiple chitinolytic (*endo*- and *exo*-acting) and previously unidentified chitin-binding domains. Reverse genetics show that ChiA and PUL-encoded proteins involved in sugar binding, import, and chitin sensing are essential for efficient chitin utilization. Surprisingly, the ChiUL encodes two pairs of SusC/D-like outer membrane proteins. Ligand-binding and structural studies revealed functional differences between the two SusD-like proteins that enhance scavenging of chitin from the environment. The combined results from this study provide insight into the mechanisms employed by Bacteroidetes to degrade recalcitrant polysaccharides and reveal important novel aspects of the PUL paradigm.

**Conclusions:**

By combining reverse genetics to map essential PUL genes, structural studies on outer membrane chitin-binding proteins, and enzymology, we provide insight into the mechanisms employed by Bacteroidetes to degrade recalcitrant polysaccharides and introduce a new saccharolytic mechanism used by the phylum Bacteroidetes. The presented discovery and analysis of the ChiUL will greatly benefit future enzyme discovery efforts as well as studies regarding enzymatic intramolecular synergism.

**Electronic supplementary material:**

The online version of this article (doi:10.1186/s13068-016-0674-z) contains supplementary material, which is available to authorized users.

## Background

The enzymatic deconstruction of carbohydrate biomass is of great importance in the global carbon cycle. Increased understanding is crucial for development of more efficient processes for enzymatic biomass conversion, which may contribute to reducing the dependency on fossil fuels in society. Chitin is one of the most abundant polysaccharides on earth, second only to cellulose, and is found primarily in fungi and the exoskeletons of arthropods. Similar to cellulose, which consists solely of β(1→4)-linked d-glucose units, chitin consists of a single type of monosaccharide, β(1→4)-linked *N*-acetyl-d-glucosamine (GlcNAc), and the insoluble nature of both polysaccharides leads to the formation of crystalline fibers which are highly recalcitrant to degradation. Enzymatic conversion of chitin typically requires multiple activities, including *endo*-acting non-processive chitinases and *exo*-acting processive chitinases that depolymerize the chains from either the reducing or the non-reducing ends. In many aerobic systems, lytic polysaccharide monooxygenases (LPMOs) also participate [1].

Bacteria belonging to the phylum Bacteroidetes have long been recognized as especially proficient carbohydrate degraders. The predominant focus on these species has been related to host-associated anaerobic Bacteroidetes that dominate the gut microbiota of mammals, including humans [2, 3], though Bacteroidetes species are common in a wide range of both aerobic and anaerobic environments [4]. Much of the carbohydrate degradation capabilities of the Bacteroidetes can be attributed to their use of polysaccharide utilization loci (PULs), which are gene clusters encoding many of the necessary functions in the binding, sensing, degradation, and import of specific carbohydrates [5]. Thus far, no LPMOs have been discovered in Bacteroidetes members. The archetypal starch utilization system (Sus) from *Bacteroides thetaiotaomicron* was the first described PUL and homologs to its tandem SusC/D pair (outer membrane porin and carbohydrate-binding protein, respectively) are now the identifiers for PULs in other organisms [6]. In addition to one or more SusC/D pairs, functional PULs contain a variable number of enzymes as well as a sugar-sensing apparatus. The SusC/D-like pairs are believed to be specific for their cognate carbohydrate targets, and act in concert to bind (SusD) and transport (SusC) oligosaccharides across the outer membrane. The starch PUL contains three enzymes: an outer membrane-bound amylase (SusG) and two periplasmic enzymes (SusA, neopullulanase, and SusB, α-glucosidase), which together enable complete degradation of starch. PULs targeting polysaccharides other than starch have recently been described and characterized, such as the xyloglucan utilization locus (XyGUL) from *B. ovatus* and yeast mannan-degrading loci from *B. thetaiotaomicron* [7, 8]. Additional PULs encoded within uncultured Bacteroidetes lineages from the rumen of herbivores have also demonstrated broad hemicellulose-degrading activities [9, 10]. As these PULs target more heterogeneous structures than the Sus, they encode a larger number of enzymes, reflecting the complexity of the target polysaccharides.

So far, only PULs degrading soluble glycans have been studied in detail, and a PUL hypothesized to degrade cellulose was discovered in a recent metagenomics study [11]; however, evidence that the PUL-containing microorganism maintains growth via cellulose degradation is currently lacking. We hereby present (to our knowledge) the first in-depth study of a PUL conferring the ability to degrade an insoluble and crystalline polysaccharide, namely chitin. The studied chitin utilization locus (ChiUL) is encoded by the soil saprophyte *Flavobacterium johnsoniae*, which is a Bacteroidetes member exhibiting gliding motility [12]. *F. johnsoniae* is able to digest a wide range of polysaccharides, which can be largely attributed to the presence of 40 verified and/or predicted unique PULs [6, 12]. While not being able to degrade cellulose, *F. johnsoniae* readily digests chitin. Previous studies have shown the enzyme ChiA (Fjoh_4555), which is encoded by the ChiUL, to be essential for chitin degradation [13]. Interestingly, ChiA is fully secreted from the cell in soluble form by the newly discovered Type IX secretion system (T9SS) [14], whereas in previously described Bacteroidetes-affiliated PULs the key *endo*-acting enzymes are anchored to the outer membrane as lipoproteins [7, 8, 15]. Through a combined effort, using biochemistry, reverse genetics, and structural biology, we have revealed how *F. johnsoniae* deploys the ChiUL-encoded multi-domain chitinase ChiA in concert with additional enzymes, surface glycan-binding proteins, porins, and regulatory proteins to efficiently metabolize the crystalline polysaccharide chitin. We here provide insight into the mechanisms employed by Bacteroidetes to degrade recalcitrant polysaccharides and reveal important novel aspects of the PUL paradigm.

## Results and discussion

**Fig. 1:**
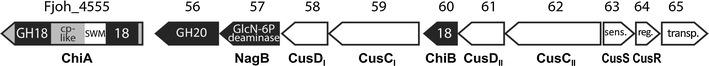
Genomic map of the ChiUL, with locus tag numbers and gene product names, where applicable; catalytic modules are in *black* with CAZy family memberships or predicted activity indicated, in the case of NagB

The ChiUL of *F. johnsoniae* consists of eleven genes that encode four enzymes, a predicted inner membrane transporter, a predicted two-component sensor/regulator system (TCS), and two individual SusC/D-like pairs (CusC/D, chitin utilization system; Fig. 1). The enzymes encoded by the ChiUL were all predicted to participate in chitin turnover, and include a multimodular chitinase (ChiA), comprising two glycoside hydrolase family 18 (GH18) domains, a second GH18 chitinase (ChiB), a GH20 *N*-acetylhexosaminidase (chitobiase), and a glucosamine-6-phosphate deaminase (NagB).

**Fig. 2:**
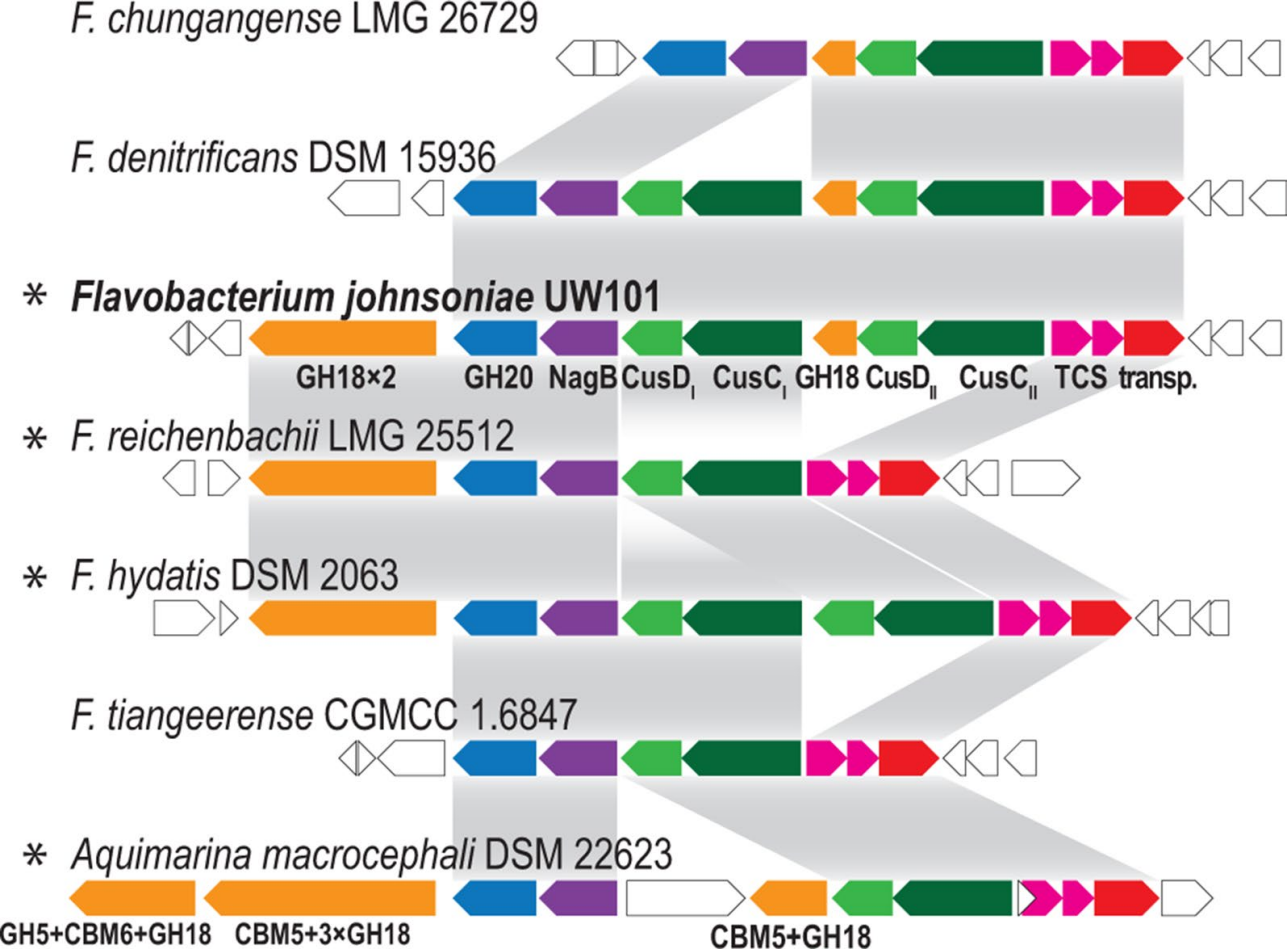
PULs with overall and partial synteny with the *F. johnsoniae* ChiUL. Color coding follows that of the labeled ChiUL genes. Homologous regions are highlighted by *gray bands*; genes with unknown function and genes flanking the loci are shown in *white*. *Asterisks* signify species able to degrade chitin. Predicted glycoside hydrolases with different modularity compared to the ChiUL genes are indicated by their CAZy family memberships

Genomic comparisons showed that homologous systems to the ChiUL occur in other Bacteroidetes members, with varying degrees of similarity (Fig. 2). In species encoding homologous PULs, the presence of a multicatalytic homolog to ChiA is directly correlated to the ability to utilize chitin (Fig. 2), though functional studies on these homologs are currently lacking.

### Disruption of enzyme-encoding genes

**Fig. 3:**
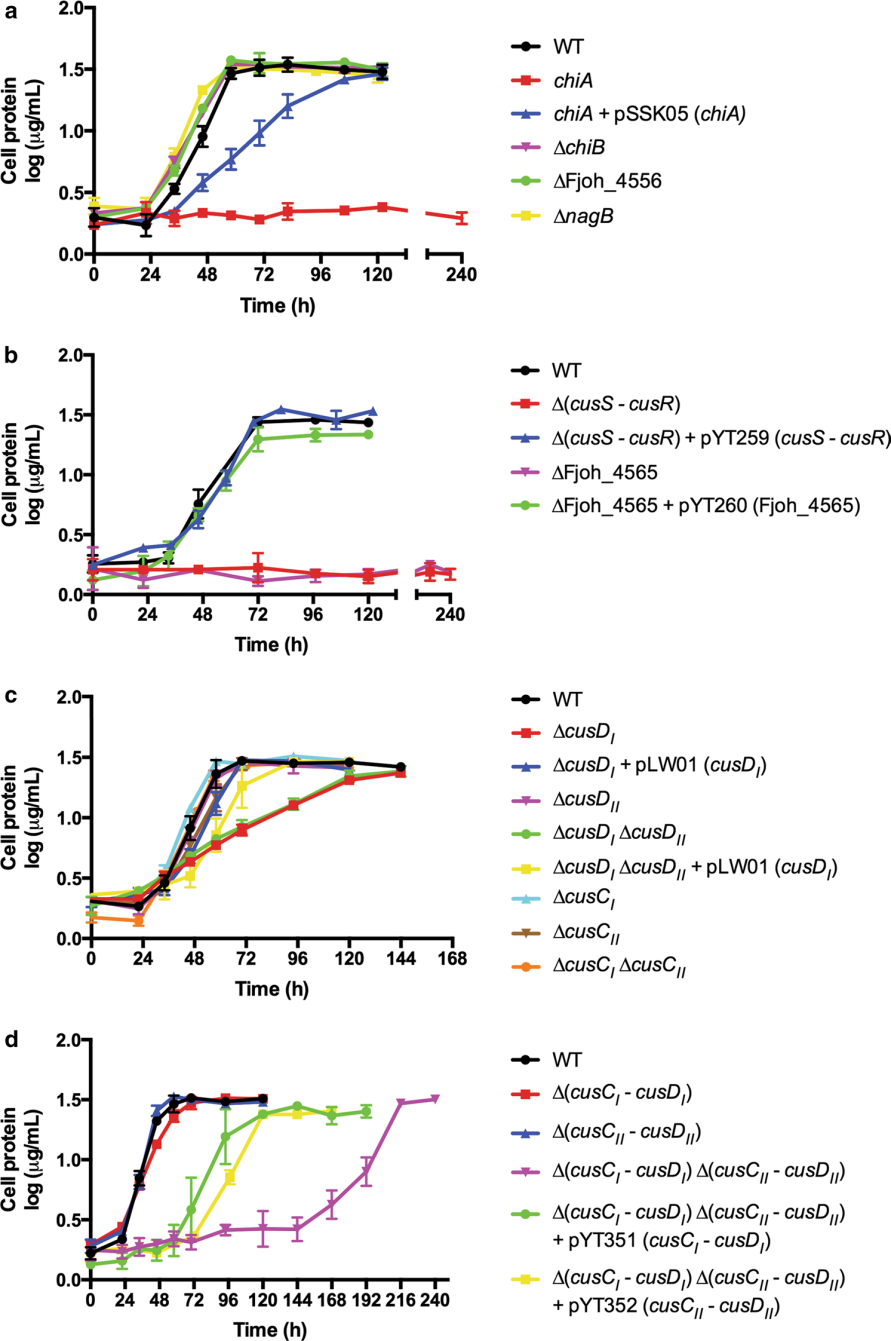
Growth curves of gene deletion mutants. **a** Mutants lacking enzymes, **b** mutants lacking the two-component regulation system and inner membrane sugar transporter, **c** mutants lacking individual CusC and CusD proteins, and mutants lacking both CusC or both CusD proteins, and** d** mutants lacking CusC/CusD pairs. pSSK05 expresses ChiA, pYT259 expresses the two-component signal transduction proteins CusS and CusR, pYT260 expresses the predicted cytoplasmic membrane sugar transporter Fjoh_4565, pLW01 expresses CusD_I_, pYT351 expresses CusC_I_ and CusD_I_, and pYT352 expresses CusC_II_ and CusD_II_. Cells (0.1 ml, OD_600_ = 1.0) were introduced into 50 ml of Stanier medium supplemented with 0.05% chitin in 250-ml flasks and incubated with shaking at 25 °C. Growth presented as log (µg cell protein/ml). Growth experiments were performed in triplicate and error bars indicate standard deviations

In order to understand the individual roles of the ChiUL gene products during growth on recalcitrant chitin crystals, we disrupted the genes of the ChiUL, to create single- and multi-gene knock-out mutants (Additional file 1: Tables S1–S3). The *chiA* disruption mutant was completely unable to grow on chitin, reaffirming the essential role of ChiA in chitin utilization (Fig. 3a; Additional file 1: Figure S1) [13]. Deletion of *chiB* or the GH20 chitobiase had no apparent effect on chitin utilization (Fig. 3a). The growth of these mutants on chitin may be hypothetically explained by redundancy, as the genome encodes other putative chitin-degrading proteins belonging to families GH18 (Fjoh_4175 and 4757), GH19 (Fjoh_2608 and 2261), and GH20 (Fjoh_0674, 2039 and 4808) [12], with signal peptides predicted for the GH18 and GH20 enzymes [16]. None of these enzymes have multiple predicted catalytic modules, and ChiA thus appears unique. Deletion of *nagB* also had no effect on chitin utilization (Fig. 3a), which may also be hypothetically explained by redundancy since the genome encodes three additional *nagB*-like genes [12].

### Disruption of other ChiUL genes

Deletion of the genes encoding the two-component regulatory system (TCS) proteins CusS (Fjoh_4563) and CusR (Fjoh_4564) abolished the ability to grow on chitin as a sole carbon source (Fig. 3b). ChiA was absent in the mutant cells and was not detected in secreted form in the spent medium (Additional file 1: Figure S2). In contrast, wild-type (wt) levels of ChiA were present in spent media of all other mutants that were defective in chitin utilization, with the exception of the *chiA* mutant (Additional file 1: Figure S2). The phenotype of the Δ(*cusS*–*cusR*) mutant was rescued by the introduction of *cusS* and *cusR* on a plasmid (Fig. 3b; Additional file 1: Figure S2). The predicted function of the TCS is to sense chitooligosaccharides (ChiOs) present in the periplasm and trigger transcription of the other ChiUL genes, similar to the role of the *B. thetaiotaomicron* SusR in starch utilization [5, 17].

PUL architectures with multiple SusC/D-like pairs have been identified but to date no detailed characterization of these systems has been performed [6]. Chitin hydrolysis only releases ChiOs, making the dual CusC/D pairs of the *F. johnsoniae* ChiUL puzzling. *cusC*_*I*_ (Fjoh_4559), *cusD*_*I*_ (Fjoh_4558), *cusC*_*II*_ (Fjoh_4562), and *cusD*_*II*_ (Fjoh_4561) were deleted individually and in combinations. ∆*cusD*_*I*_ was the only single-gene mutant and ∆(*cusD*_*I*_/*cusD*_*II*_) the only double-gene mutant with a negatively affected phenotype, exhibiting identical growth defects (Fig. 3c), with cells growing slower on chitin compared to wt cells, but with a similar final biomass. The growth defects of both ∆*cusD*_*I*_ and ∆(*cusD*_*I*_/*cusD*_*II*_) were rescued by the introduction of *cusD*_*I*_ on a plasmid, which verified the involvement of *cusD*_*I*_ in chitin utilization. Notably, the unexpected lack of phenotype for ∆(*cusC*_*I*_/*cusD*_*I*_) suggests that the growth defects in the Δ*cusD*_*I*_ mutants may have been caused by detrimental effects of CusC_I_ being produced without its CusD_I_ partner (Fig. 3d). Indeed, we observed that mild overexpression of CusC_I_ in wild-type *F. johnsoniae* hampered growth on chitin (Additional file 1: Figure S3A). This was not the result of a general growth defect since the expression of CusC_I_ had no effect on growth on glucose (Additional file 1: Figure S3B). We currently have no explanation for this observation, but future studies may reveal aspects on the interactions between SusC-like and SusD-like proteins.

SusC in *B. thetaiotaomicron* is essential for starch utilization [18], and the lack of *cusC* mutant phenotypes was therefore unexpected. The genome of *F. johnsoniae* encodes 44 predicted SusC-like proteins [12], which possibly means there is functional overlap with CusC_I_ and/or CusC_II_. Elimination of both *cusC/D* pairs yielded cells that were severely crippled for growth on chitin, requiring five times as long to reach the final biomass of wt cells (Fig. 3d). The growth defect was partially restored by plasmids carrying *cusC*_*I*_–*cusD*_*I*_ or *cusC*_*II*_–*cusD*_*II*_, respectively (Fig. 3d). Cells of the [Δ(*cusC*_*I*_–*cusD*_*I*_) Δ(*cusC*_*II*_–*cusD*_*II*_)] mutant grew as well as wt cells on glucose or GlcNAc, and thus did not have a general growth defect (Additional file 1: Figure S4). Taken together, the data from the knock-out strains suggest that at least one of these pairs is needed for efficient growth on chitin. A mutant lacking the predicted inner membrane sugar transporter (major facilitator superfamily MFS_1) Fjoh_4565 was unable to grow on chitin (Fig. 3b). It also failed to grow on GlcNAc or glucose (Additional file 1: Figure S4), suggesting a role in transport of these monosaccharides into the cytoplasm. The growth defects were rescued by the introduction of the gene on a plasmid (Fig. 3b; Additional file 1: Figure S4). Although the mutant failed to grow on chitin, GlcNAc, and glucose, it grew as well as the wild type on peptides (CYE growth medium, Additional file 1: Figure S4) indicating that it did not have a general growth defect.

### Binding profiles and structures of the SusD-like proteins, CusD_I_ and CusD_II_

CusD_I_ and CusD_II_ exhibit comparatively low sequence identity (27%) and may thus have different carbohydrate affinity characteristics. Isothermal calorimetry (ITC) studies with the recombinantly produced proteins revealed that CusD_I_ binds the full range of tested oligomers (chitobiose to chitohexaose) with similar affinities, and even GlcNAc, though with much lower affinity (Table 1). In this assay, CusD_I_ displayed some enhanced affinity for chitotriose. The precise reason for this is unclear, although it is likely a minor artifact of fitting the data to a one-site binding model in which n (stoichiometry) and *K*_D_ were determined from the experimental data; an apparent trend here is that lower *n* values correspond somewhat with lower *K*_D_ values. However, it is also possible that chitotriose is a somewhat more ideal ligand because the reducing and non-reducing ends of the oligosaccharide make fortuitous hydrogen-bonding interactions with the protein. In contrast, CusD_II_ only bound chitopentaose and chitohexaose, with 10-fold higher affinity for the latter. It is likely that both CusD proteins are able to bind even longer chitin fragments, which due to solubility issues are not amenable to ITC analysis. To determine whether CusD_I_ and CusD_II_ displayed a preference for crystalline chitin, a pull-down study was performed. Both proteins showed a clear binding preference to chitin over cellulose (Avicel) and starch (amylopectin), respectively, indicating that chitin polysaccharides may be bound by the proteins in vivo (Additional file 1: Figure S5).

**Table 1:** Summary of the dissociation constants and stoichiometry obtained by isothermal titration calorimetry for the binding of chitooligosaccharides to the SusD-like proteins

Protein	Substrate	K_D_ (µM)	*n*
CusD_I_	GlcNAc	1470 ± 210	1^a^
	Chitobiose	25.6 ± 2.3	1.21 ± 0.07
	Chitotriose	8.51 ± 0.51	0.88 ± 0.08
	Chitotetraose^b^	38.3 ± 4.3	1.19 ± 0.03
	Chitopentaose	12.8 ± 0.21	0.79 ± 0.02
	Chitohexaose	16.1 ± 0.42	0.98 ± 0.06
CusD_II_	Chitopentaose	236 ± 8	1.65 ± 0.07
	Chitohexaose	41.2 ± 1.0	1.50 ± 0.29

Error margins represent standard errors of the mean from 2–3 titration experiments unless otherwise noted

^a^ *n* value was constrained to 1 in a one-site binding model; see “Methods” section

^b^Single titration performed; errors are displayed for the fitting to a one-site binding model

In order to investigate the molecular basis of this differential ChiO binding, we determined the crystal structures of CusD_I_ (1.4 Å resolution, *R*_w_ = 15.9% *R*_f_ = 17.6%, PDB accession code 5J90, Additional file 1: Table S4) and CusD_II_ (2.3 Å resolution, *R*_w_ = 21.4%, *R*_f_ = 26.3%, PDB accession code 5J5U, Additional file 1: Table S4). Despite considerable efforts, crystals with bound ChiO ligands were not obtained, likely due to crystal packing, which for both proteins affected the predicted ligand-binding site.

**Fig. 4:**
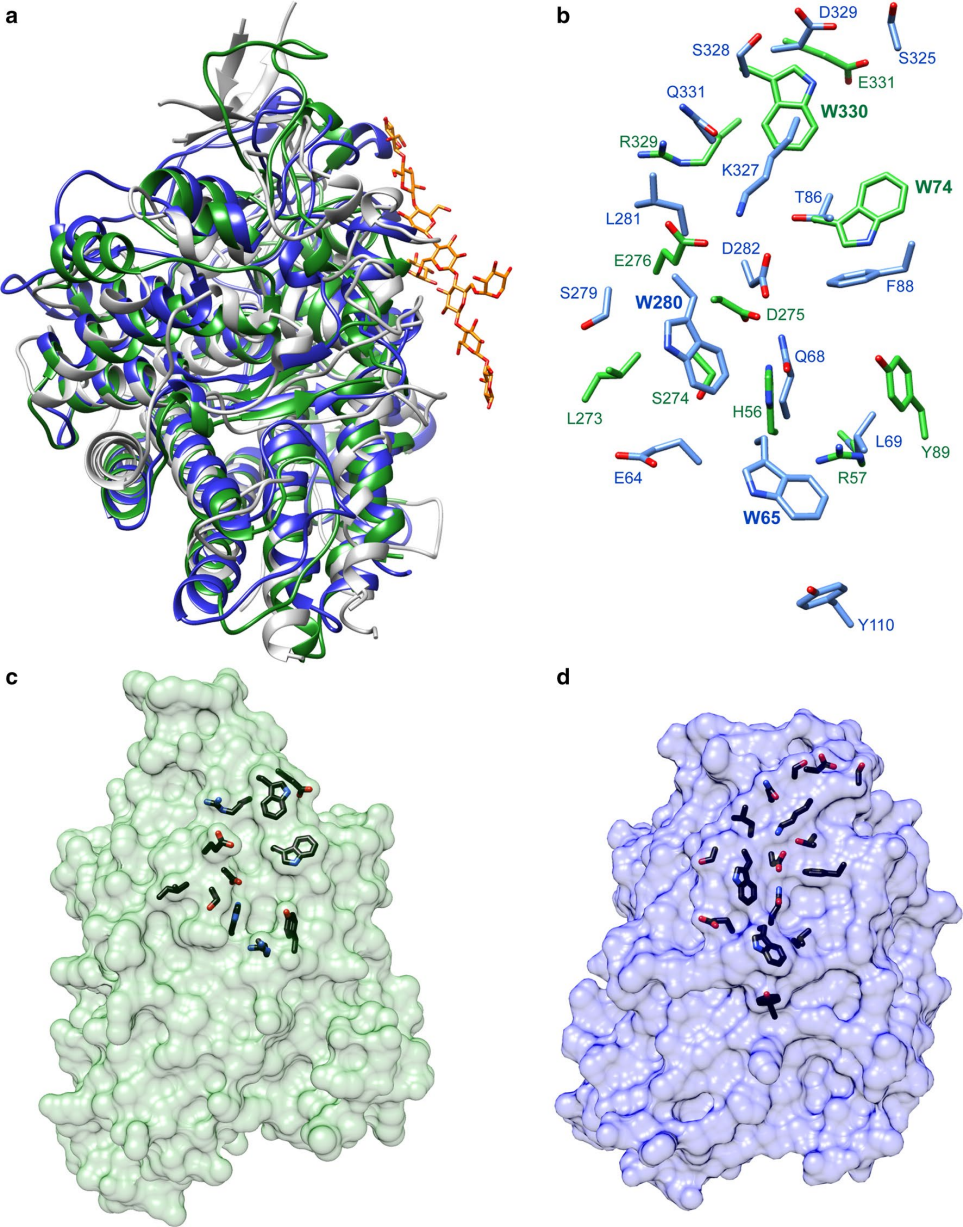
Molecular structures of CusD_I_ and CusD_II_. **a** Overlay of ribbon representations of CusD_I_ (*blue*), CusD_II_ (*green*) with *B. ovatus* SusD homologue SGBP-A (*gray*, PDB 5E76). Xyloglucooligosaccharide (XyGO_2_) bound to SGBP-A is shown in *yellow* and *red sticks*. **b** Surface-accessible residues of CusD_I_ (*blue*) and CusD_II_ (*green*) within 5 Å of the XyGO_2_ from the superposition with SGBP-A are displayed. **c** and **d** Space-fill models of CusD_I_ and CusD_II_, respectively, with surface-accessible residues, as in **b**, in *black*, illustrating the different binding surfaces of the two proteins. Surface accessibility was calculated using CCP4 (40)

The overall structures of CusD_I_ and CusD_II_ overlay with an RMSD of 2.2 Å for 363 C*α* pairs and display the canonical SusD protein fold featuring four tetratricopeptide repeats that act as a structural scaffold (Fig. 4a) [19]. To determine the predicted glycan-binding site of both proteins, we superimposed these with the structures of the *B. thetaiotaomicron* SusD in complex with maltoheptaose (PDB 3CK9) and SGBP-A (PDB 5E76), a SusD-like protein from *B. ovatus*, in complex with a xyloglucan fragment. Based on the conserved structures and glycan-binding sites in both SusD and SGBP-A, we predict that the CusD glycan-binding sites are located in similar positions. The most striking feature of the putative substrate-binding regions of both CusD_I_ and CusD_II_ is the presence of two Trp residues that would provide a flat platform for binding ChiOs as well as insoluble chitin, as has been observed in chitin-binding carbohydrate-binding modules (CBMs) [20, 21]. CusD_I_ features W280 and W65, whereas CusD_II_ displays W330 and W74 that are located at non-equivalent positions but may nevertheless have similar roles (Fig. 4b–d). While both glycan-binding platforms overlay within the known glycan-binding site of SGBP-A, they are offset from each other, suggesting that ChiOs are bound differently by each protein. The area of CusD_I_ surrounding W280 and W65 displays several residues that may provide hydrogen-bonding or electrostatic interactions with individual GlcNAc residues of chitin, while there are fewer such residues within the aromatic interface of CusD_II_. This may account for the difference in binding specificity and affinity between the two proteins. Note that for both proteins the Trps provide a flat platform on the surface of the proteins that would support binding to chitin (Fig. 4c, d).

### The ChiUL chitinases

ChiA is an extracellularly localized 169-kDa enzyme, which consists of two individual GH18 domains, located near the N- and C-termini of the protein (ChiA_N, ChiA_C, Fig. 1), whereas ChiB is a single-GH18 domain enzyme. The *F. johnsoniae* GH18 domains were all similar to different members of the well-characterized chitinolytic system from *Serratia marcescens*, with ChiA_N being most similar to *Sm*ChiA, an *exo*-chitinase (27% identity), ChiA_C most similar to *Sm*ChiC, an *endo*-chitinase (27% identity), and ChiB most similar to *Sm*ChiB, an *exo*-chitinase (25% identity). The presence of a so-called *α*+*β* insertion domain between strand 7 and helix 7 of the (*α*/*β*)_8_-barrel of the catalytic domain is associated with a higher degree of *exo*-character [1]. This domain is missing in ChiA_C, while a long variant is present in ChiA_N and a shorter variant occurs in ChiB (Additional file 1: Figure S6). Together, these observations suggest that the various GH18 domains in the ChiUL have different functions.

### ChiA—an essential, secreted and multimodular chitinase

ChiA was produced both as an intact 155.5-kDa protein (ChiA_F; residues 26–1475), lacking the N- and C-terminal signal peptides (the latter allowing for T9SS secretion [14]), and in truncated forms. The 49.5-kDa ChiA_N (residues 26–446) and 40.7 kDa ChiA_C (residues 1108–1475) GH18 domains, as well as the 70.3-kDa region between the GH18 domains, ChiA_M (residues 464–1137), were expressed individually. Unexpectedly, ChiA_N and ChiA_C displayed different pH optima (Additional file 1: Figure S7), with ChiA_N performing best at pH 4 and ChiA_C at pH 6.5. ChiA_N and ChiA_C hydrolyzed ChiOs from chitotriose to chitohexaose, with a preference for longer substrates (Table 2). The *k*_*cat*_ */K*_m_ values for both enzymes were several orders of magnitude lower for chitotriose than for the best substrates, suggesting that extensive substrate binding is necessary for full activity. ChiA_N released only chitobiose and chitotetraose from chitohexaose, which, together with the presence of a large so-called *α*+*β* domain suggests an *exo*-acting and likely processive character. On the other hand, ChiA_C also released chitotriose and had generally lower *k*_*cat*_ */K*_m_ values, which, together with the absence of an *α*+*β* domain, suggests an *endo*-acting character [22].

**Table 2:** Summary of catalytic parameters for the ChiUL enzymes

Enzyme	Substrate	*k*_*cat*_ (s^−1^)	*K*_m_ (mM)	*k*_*cat*_ */K*_m_ (mM^−1^ s^−1^)
ChiA_N	Chitotriose	0.12 ± 0.007	4.0 ± 0.4	0.031
	Chitotetraose	17.4 ± 0.65	0.050 ± 7.3 × 10^−3^	349
	Chitopentaose	20.7 ± 0.93	0.017 ± 3.9 × 10^−3^	1190
	Chitohexaose^a^	48.8 ± 6.1	0.051 ± 0.012	951
ChiA_C	Chitotriose	0.014 ± 6.0 × 10^−4^	1.17 ± 0.15	0.012
	Chitotetraose	0.99 ± 0.03	1.11 ± 0.08	0.89
	Chitopentaose	1.66 ± 0.047	0.15 ± 0.02	11.1
	Chitohexaose	1.29 ± 0.022	0.071 ± 3.5 × 10^−3^	18.3
ChiB	Chitotriose	0.39 ± 0.014	5.6 ± 0.40	0.069
	Chitotetraose^a^	9.77 ± 1.56	2.2 ± 0.45	4.4
	Chitopentaose^a^	19.9 ± 1.48	0.40 ± 0.052	49.4
	Chitohexaose^a^	60.1 ± 7.4	0.45 ± 0.08	135
GH20	Chitobiose	164 ± 10	0.83 ± 0.13	198
	Chitotriose	113 ± 6.8	0.65 ± 0.11	175
	Chitotetraose	106 ± 4.1	0.66 ± 0.072	162
	Chitopentaose	104 ± 2.8	0.61 ± 0.048	170
	Chitohexaose	94.2 ± 4.1	0.51 ± 0.066	183

Values represent means and standard errors from duplicate experiments

Data were fitted to the Michaelis–Menten or substrate inhibition equations (*v*_*o*_ = *V*_max_*[*S*]/(*K*_m_ + [*S*] × (1 + [*S*]/*K*_*i*_)))

^a^Reactions for which substrate inhibition was observed

**Fig. 5:**
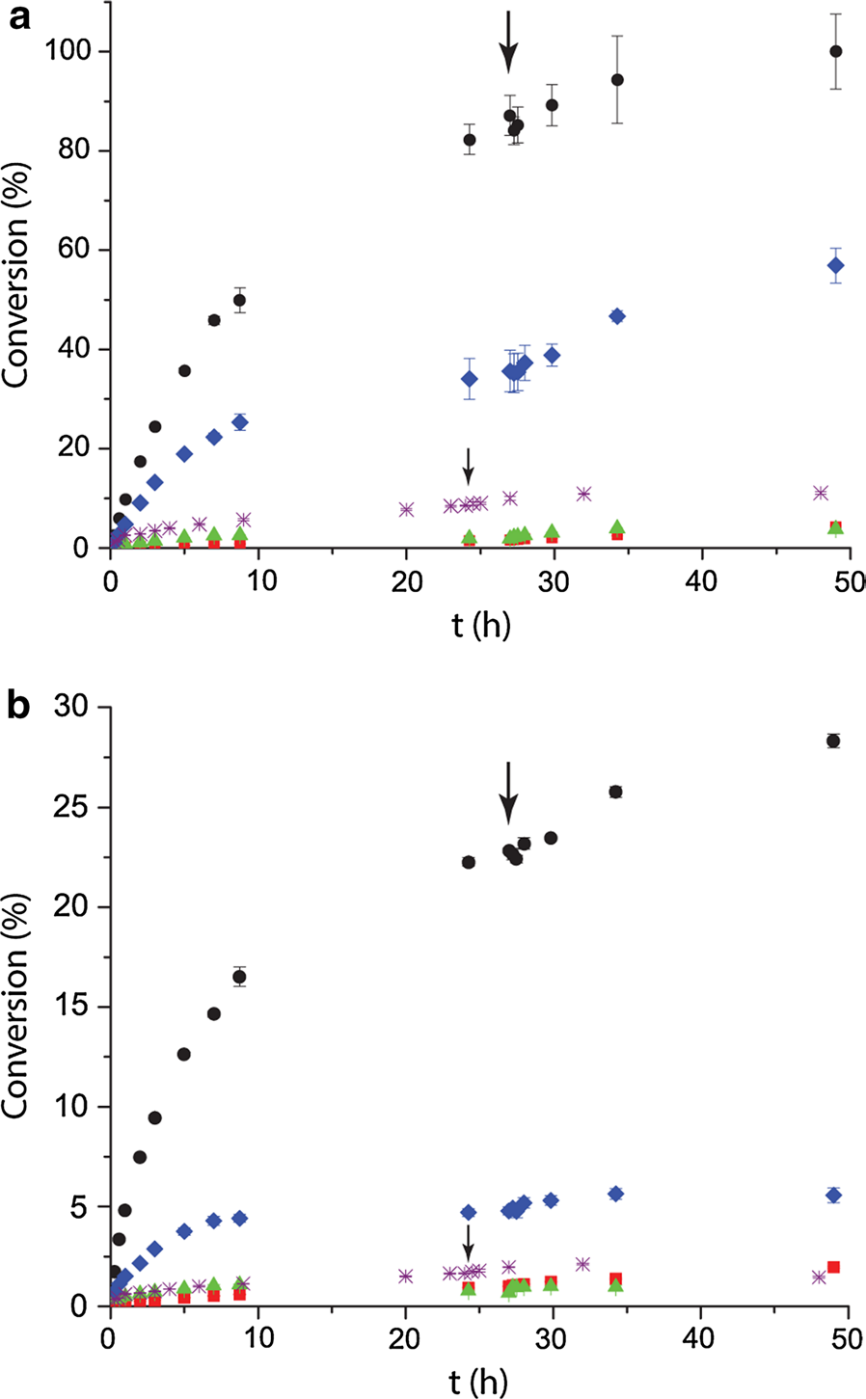
Chitin degradation by the ChiUL. The ChiUL-encoded chitinase enzymes were incubated with β-chitin (**a**) or α-chitin (**b**) at 5 g/l, respectively. Enzyme reactions are labeled as follows: *black circles*—ChiA_F (1 µM), *red squares*—ChiA_N (10 µM), *green triangles—*ChiA_C (10 µM), *purples crosses—*ChiB (10 µM), and *blue diamonds*—synergy reaction (ChiA_N & ChiA_C; 5 µM each). Additional enzyme was added after 27 h, indicated by large arrows (24 h for ChiB, *small arrows*). No observable hydrolytic activity could be detected by ChiA_M. Data points are the average of triplicate experiments and *error bars* indicate standard errors

The activities of the three enzyme versions of ChiA were evaluated on crystalline α- and β-chitin, of which the former is more recalcitrant. ChiA_F hydrolyzed over 80% of β-chitin within 24 h (1 µM enzyme, 5 g/l substrate, pH 6.5), and close to 25% of α-chitin in identical conditions (Fig. 5), producing mainly chitobiose and lower amounts of GlcNAc. The rate of chitin turnover is comparable to or better than how the well-studied *S. marcescens* GH18 chitinases ChiA, ChiB, and ChiC in a synergistic manner have been shown to degrade approximately 50% of β-chitin over 24 h (each enzyme at 50 nM, 0.1 g/l β-chitin, 37 °C) [23]. Chitin degradation by the individual GH18 domains was very poor compared to ChiA_F. At ten times higher enzyme concentration (10 µM), ChiA_N and ChiA_C converted 1.5–2% of β-chitin and less than 1% of α-chitin in 24 h, respectively. A strong synergistic effect could however be observed when the enzymes were combined, yielding 35 and 5% conversion of β- and α-chitin, respectively (5 µM of each enzyme), values that notably are still much lower than those obtained with ChiA_F (at 1 µM). Addition of more enzyme after 27 h did not alter the conversion rate in ChiA_F reactions, but gave a slight activity boost in the single domain and synergy reactions, indicating lower stability than ChiA_F or enzyme inactivation.

**Fig. 6:**
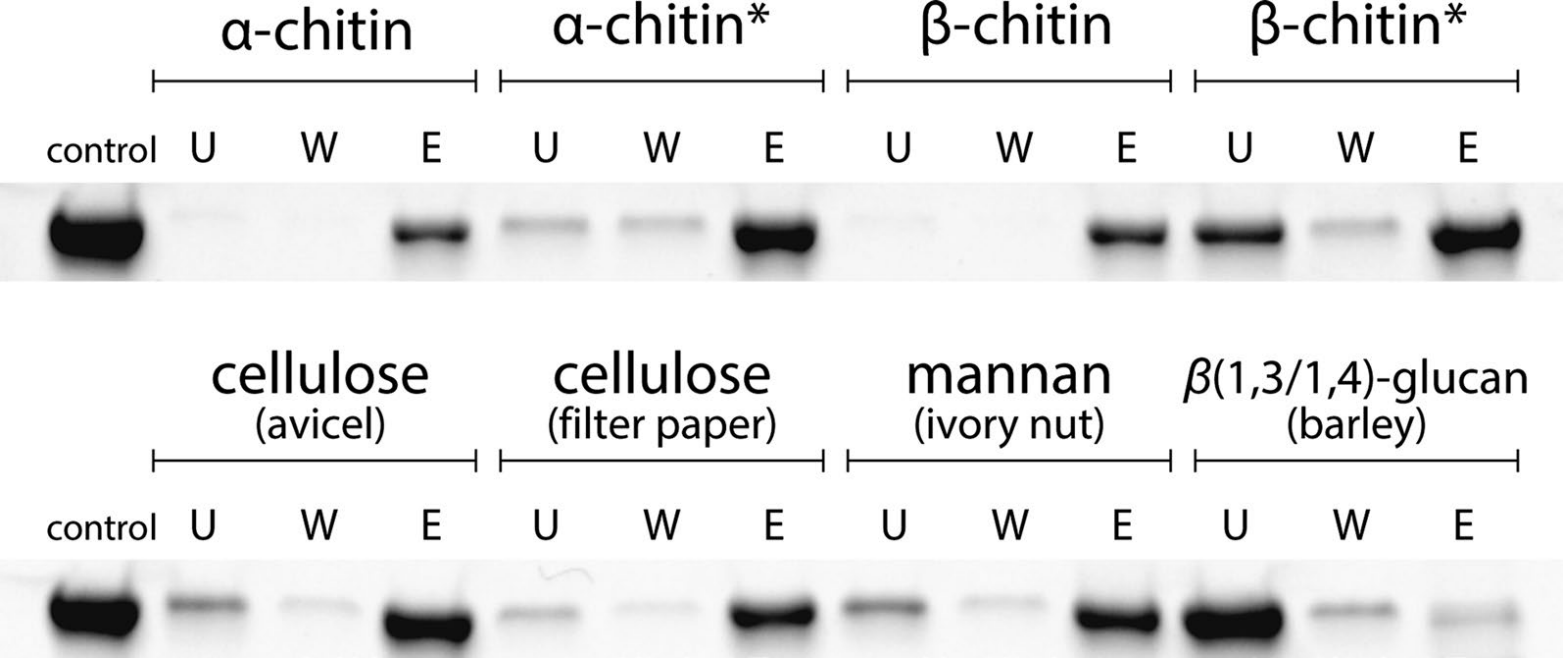
Binding analysis of ChiA_M to insoluble polysaccharides. *Lanes* control, protein without substrate, *U* unbound protein, *W* wash fraction, *E* proteins eluted by incubation with urea. *Lanes* marked with an *asterisk* in the *upper row* refer to experiments using ball-milled chitin, while unmarked lanes refer to crushed chitin, sieved to a particle size of ≤0.85 mm

The multimodular architecture of ChiA is strikingly similar to that of the *Caldicellulosiruptor bescii* CelA cellulase, shown to perform as well as complex commercial enzyme cocktails in cellulose turnover [24]. CelA comprises an N-terminal GH9 *endo*-glucanase, three CBMs, and a C-terminal GH48 *exo*-cellulase [24]. The internal ‘middle’ region of ChiA, ChiA_M, was not predicted to contain either carbohydrate-active enzymes or CBMs. Instead, five carboxy-peptidase regulatory-like domain repeats (Pfam13620, residues ~470–890) followed by an SWM_repeat domain (Pfam13753, putative flagellar system-associated, residues ~900–1100) were predicted. We produced ChiA_M (residues 464–1137) in order to clarify its role in ChiA. Neither chitinolytic nor proteolytic activity was observed. Instead, apparent binding to crystalline substrates, i.e., α- and β-chitin, cellulose (Avicel and milled filter paper), and insoluble ivory nut mannan (Fig. 6) was observed. The protein did not bind to the insoluble fraction of barley β-glucan, plausibly as the kinks in its structure prevents the formation of crystallites. The binding of ChiA_M to larger chitin particles was especially strong, with only a minor fraction of the total bound protein released after incubation with 8 M urea (Fig. 6).

### ChiB and the chitobiase—non-essential enzymes active on ChiOs

Similar to ChiA_N and ChiA_C, ChiB was active on ChiOs with a preference for longer substrates (Table 2). The hydrolysis of chitohexaose produced equal amounts of chitotriose and chitobiose/chitotetraose, indicating an *endo* mode of action. The enzyme was much less efficient in deconstructing crystalline chitin than ChiA_F, converting ~10 and 2% of β- and α-chitin, respectively (24 h, 10 µM enzyme). As discussed earlier, homologues to *chiB* are found in PULs from bacteria unable to utilize chitin as the sole carbon source, and oligosaccharides rather than crystalline chitin may be the main substrate for ChiB in vivo. ChiB is a predicted lipoprotein and likely resides on the cell surface similar to typical *endo*-acting lipo-tagged PUL enzymes [5, 7, 8]. The enzyme is not essential for chitin degradation and may act on products released by ChiA. The GH20 chitobiase encoded by the ChiUL rapidly hydrolyzed all ChiOs tested, at similar rates, into GlcNAc (Table 2). The enzyme is predicted to be periplasmic, in keeping with the general mechanism of PULs where the final degradation into monosaccharides occurs in the periplasm. Despite our efforts, we were unable to express the predicted glucosamine-6-phosphate deaminase (NagB) in *E. coli*, and its predicted biological role in conversion of glucosamine-6P to fructose-6P could thus not be explored.

## Concluding remarks

**Fig. 7:**
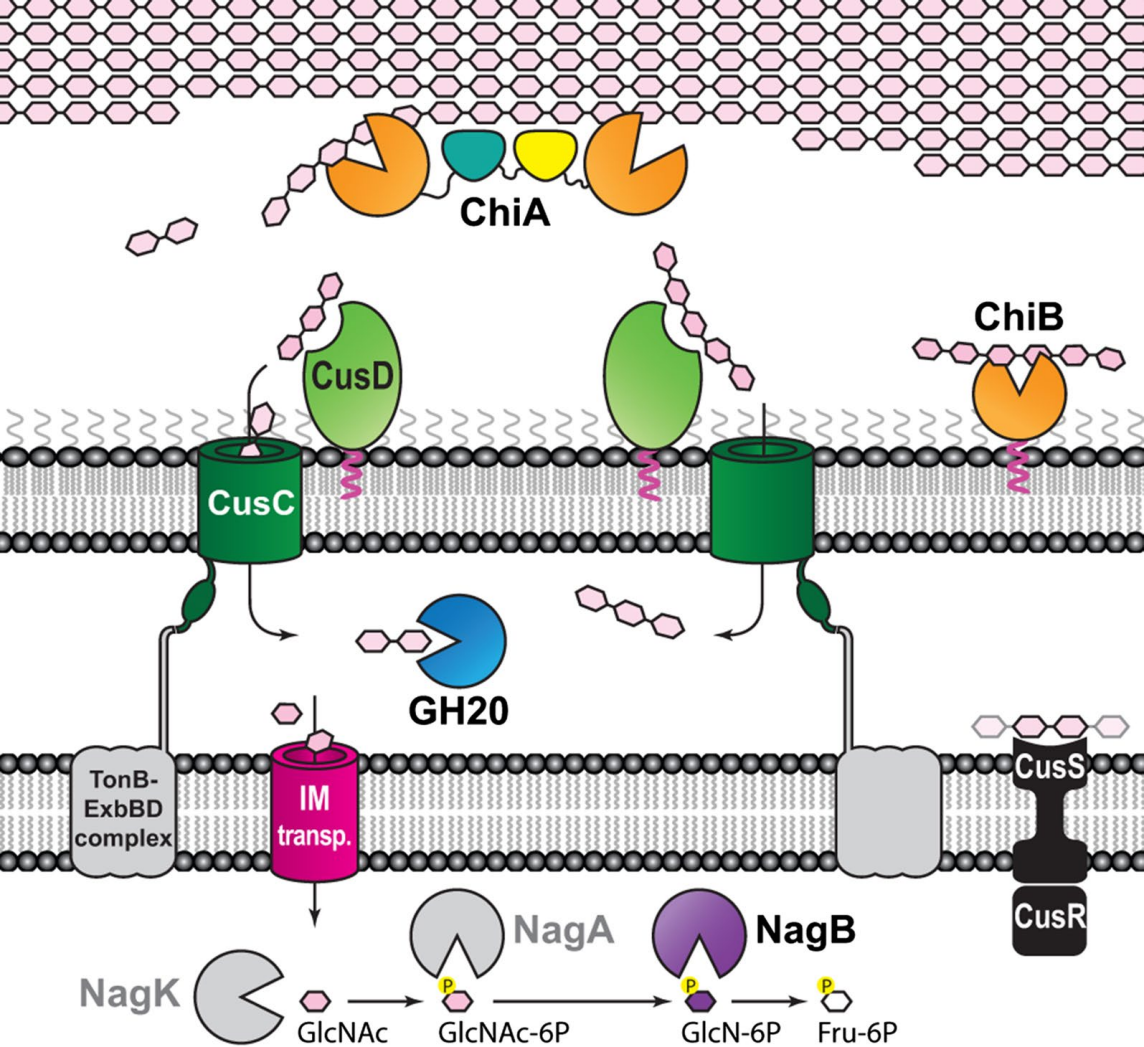
The proposed pathway of chitin degradation by the ChiUL. ChiA is fully secreted from the cell and hydrolyzes chitin by the combined actions of the N- and C-terminal GH18 domains and the carbohydrate-binding properties of ChiA_M. ChiB, while not as efficient as ChiA, hydrolyzes chitin and chitooligosaccharides (ChiOs) at the cell surface. The dual CusC/D pairs capture and import ChiOs into the periplasm, where the GH20 enzyme fully degrades them into monosaccharides. After import into the cytoplasm, NagK (Fjoh_4589), NagA (Fjoh_3974), and NagB are predicted to convert N-acetyl-glucosamine to fructose-6-phosphate

Until now, little was known about chitin conversion by *F. johnsoniae* and, indeed, of the conversion of any insoluble polysaccharide by members of the phylum Bacteroidetes. We show here that crystalline chitin conversion by *F. johnsoniae* correlates with the presence of the ChiUL, and have determined the key proteins involved in the chitinolytic process by this bacterium. Our results allow us to propose a complete model of chitin utilization by this bacterium (Fig. 7), which commences with the action of ChiA on crystalline chitin polysaccharides. The resulting ChiOs, and likely chitin crystals as well (analogous to the archetypal starch-binding SusD protein of *B. thetaiotaomicron*), are captured by the CusD proteins. Soluble oligosaccharides are imported into the periplasm and fully hydrolyzed into monosaccharides for further metabolism. In keeping with previously studied PULs, the TCS sensor/signal transduction domain is predicted to trigger upregulation of the ChiUL genes upon binding chitin degradation products, thus enabling a specific and rapid response to the presence of chitin in the cell’s surroundings.

*C. bescii* (Gram-positive, thermophilic, anaerobic, cellulolytic) and *F. johnsoniae* (Gram-negative, mesophilic, aerobic, chitinolytic) have distinctly different habitats and evolutionary backgrounds. Nevertheless, the two organisms have evolved similar extracellular enzymes with multimodular architectures to efficiently disassemble recalcitrant crystalline substrates [24, 25]. *C. bescii* CelA is known for its extraordinary capacity to degrade cellulose [24], and *F. johnsoniae* ChiA has analogous properties where both enzymes use a combination of *exo*- and *endo*-acting enzyme domains and carbohydrate affinity structures on the same polypeptide chain. The synergistic complementarity of the enzyme domains may be a result of an enhanced ‘proximity effect,’ where intimate contact between the *endo*- and *exo*-glycanase domains is assured by the covalent linker that connects them, analogous to the enzyme cooperativity observed in cellulosomes [26]. Given the difference in cell architecture of Gram-negative and Gram-positive bacteria, and the lack of PULs in the latter, it is perhaps not surprising that the ChiUL-encoded sugar-binding and transport mechanisms employed by *F. johnsoniae* are not similar to the ABC transporters used by *Caldicellulosiruptor* species [27].

The combined results of the study of the ChiUL, as well as previous studies on ChiA [13], suggest a model of the chitin utilization pathway in *F. johnsoniae* (Fig. 7). The data presented here bring a new level to the understanding of how the sophisticated PUL systems of the Bacteroidetes may operate. The fact that the main chitinase of the ChiUL is fully secreted from the cell in soluble form is a mechanism of Bacteroidetes-affiliated PULs that has not been described before, and the multimodularity and catalytic power of the secreted enzyme adds another novel element to the findings. Similar multicatalytic enzyme architectures seem to be promising targets in future studies regarding deconstruction of complex biomass, and are likely a key feature to look for when scouting for novel PULs able to target highly recalcitrant polysaccharides.

## Methods

MilliQ water was used in all experiments. Chitooligosaccharides were purchased from Megazyme (Wicklow, Ireland). For growth studies, chitin powder (practical grade from crab shells; Sigma Chemical Co., St. Louis, Mo.) was prepared as a slurry essentially as described previously [28]. For enzymatic assays, the following chitin types were used: shrimp shell α-chitin was a gift from Chitinor AS (Senjahopen, Norway) and had been pretreated using standard conditions (demineralized by HCl and deproteinized by concentrated NaOH); squid pen β-chitin was a gift from Yaegaki Co. Ltd., Japan, and was prepared in a similar manner. Chitin crystals used in hydrolysis reactions were milled to ~200 µM size using a Retsch PM 100 planetary ball mill at 450 rpm using zirconium oxide balls in zirconium oxide vessels.

### Bacterial strains, plasmids, and growth conditions

*Flavobacterium johnsoniae* ATCC 17061^T^ strain UW101 was the wild-type (wt) strain used in this study [12, 29]. The streptomycin-resistant *rpsL* mutant of UW101 (CJ1827) was used to construct deletion mutants [30]. *F. johnsoniae* strains were grown in casitone yeast extract (CYE) medium at 30 °C [31] unless indicated otherwise. *Escherichia coli* strains were grown in lysogenic broth medium (LB) at 37 °C [32]. Strains, plasmids, and primers used in this study are listed in Tables S1, S2 and S3, respectively (Additional file 1). Antibiotics were used at the following concentrations when needed: ampicillin, 100 µg/ml; chloramphenicol, 30 µg/ml; erythromycin, 100 µg/ml; kanamycin, 50 µg/ml; streptomycin, 100 µg/ml; and tetracycline, 20 µg/ml.

### Construction and complementation of gene deletion mutants

Unmarked deletions were generated as previously described [30]. To delete *cusD*_*I*_ (Fjoh_4558), a 1.7-kbp fragment downstream of and spanning the final 75 bp of *cusD*_*I*_ was amplified using Phusion DNA polymerase (New England BioLabs, Ipswich, MA, USA) and primers 1166 (engineered *Xba*I site) and 1167 (engineered *Sal*I site). The fragment was digested with *Xba*I and *Sal*I and cloned into pRR51 that had been digested with the same enzymes, generating pSSK13. A 1.7-kbp fragment upstream of and spanning the first 51 bp of *cusD*_*I*_ was amplified using primers 1168 (engineered *Sal*I site) and 1169 (engineered *Sph*I site). The fragment was digested with *Sal*I and *Sph*I and fused to the downstream region of *cusD*_*I*_ by ligation with pSSK13, which had been digested with the same enzymes, to generate the deletion construct pSSK18. pSSK18 was introduced into the *F. johnsoniae* strain CJ1827 by triparental conjugation [33]. Colonies containing the plasmid integrated into the chromosome by homologous recombination were obtained by selecting for erythromycin resistance, and *cusD*_*I*_ deletion mutants that had lost the integrated plasmid were obtained by subsequently selecting for streptomycin resistance and erythromycin sensitivity, and confirmed by PCR, essentially as previously described [30].

Strains with deletions in Fjoh_4556 (GH20 chitobiase), *nagB* (Fjoh_4557), *cusC*_*I*_ (Fjoh_4559), *chiB* (Fjoh_4560), *cusD*_*II*_ (Fjoh_4561), *cusC*_*II*_ (Fjoh_4562), Fjoh_4565, and strains with deletions spanning the adjacent genes (*cusC*_*I*_–*cusD*_*I*_), (*cusC*_*II*_–*cusD*_*II*_), and *cusS*–*cusR* (Fjoh_4563–Fjoh_4564) were constructed in the same way, using the plasmids and primers listed in Tables S2 and S3 (Additional file 1), respectively. Strains with multiple deletions (∆*cusC*_*I*_ ∆*cusC*_*II*_), (∆*cusD*_*I*_ ∆*cusD*_*II*_), and (∆[*cusC*_*I*_–*cusD*_*I*_] ∆[*cusC*_*II*_–*cusD*_*II*_]) were constructed by sequential use of these procedures.

For complementation of the ∆*cusD*_*I*_ and (∆*cusD*_*I*_ ∆*cusD*_*II*_) mutants, a 1937-bp region spanning *cusD*_*I*_ was amplified using primers 1871 (engineered *Kpn*I site) and 1515 (engineered *Sph*I site) and cloned into pCP23, to generate pLW01, which was introduced into the ∆*cusD*_*I*_ and (∆*cusD*_*I*_ ∆*cusD*_*II*_) mutants by triparental conjugation. For complementation of the [∆(*cusC*_*I*_–*cusD*_*I*_) ∆(*cusC*_*II*_–*cusD*_*II*_)] mutant, a 5132-bp region spanning *cusC*_*I*_ *–cusD*_*I*_ amplified using primers 1955 (engineered *Kpn*I site) and 1956 (engineered *Sph*I site) and a 5113-bp region spanning *cusC*_*II*_ *–cusD*_*II*_ amplified using primers 1512 (engineered *Kpn*I site) and 1515 (engineered *Sph*I site) were cloned into pCP23 to generate pYT351 and pYT352, respectively. Similarly, ∆(*cusS*–*cusR*) and ∆Fjoh_4565 mutants were complemented by cloning the wt genes into pCP23 as described above, except that primers specific to each gene were used (Additional file 1: Table S3), resulting in the complementation plasmids listed in Table S2 (Additional file 1).

### Growth of *F. johnsoniae* on chitin and monosaccharides

Cells from freezer stocks were propagated on CYE agar at 30 °C for 2 days and were restreaked on fresh CYE agar and incubated at 30 °C for 1 day. Cells were scraped off the plates, suspended in 1 ml Stanier medium (1.0 g/L KNO_3_, 1.0 g/L K_2_HPO_4_, 0.2 g/L MgSO_4_·7H_2_O, 0.1 g/L CaCl_2_·2H_2_O, 0.02 g/L FeCl_3_·6H_2_O, pH 7.3) [34], and pelleted by centrifugation at 4200×*g* for 3 min to remove residual CYE medium. The cells were suspended in Stanier medium to a concentration (OD_600_) of 1.0 and used to inoculate various media.

To measure growth on chitin, 0.1 ml of the inoculation cell suspension was introduced into 50 ml of Stanier medium supplemented with 0.05% (w/v) chitin in 250-ml flasks and incubated at 25 °C with shaking (200 rpm). At various times, 1-ml samples were removed. Cells and residual chitin were collected by centrifugation at 17,000×*g* for 10 min. Growth was assessed by determining total cellular protein in the pellets as previously described [35]. To observe the utilization of chitin and cell growth simultaneously, 0.02 ml of the inoculation cell suspension was introduced into 10 ml of Stanier medium supplemented with 0.05% (w/v) chitin in 150 mm by 25 mm test tubes and incubated at 25 °C with shaking. Test tubes were photographed at various times to monitor chitin levels and turbidity (cell growth).

To measure growth on glucose or N-acetyl-glucosamine (GlcNAc), 0.1 ml of the inoculation cell suspension was introduced into 50 ml of Stanier medium supplemented with 0.1% (w/v) glucose or GlcNAc in 250-ml side-arm flasks and incubated at 25 °C with shaking. Turbidity was monitored using a Klett-Summerson photoelectric colorimeter (Klett Mfg. Co., NY, USA). Growth experiments were performed in triplicate.

### Western blot immunodetection of ChiA

*F. johnsoniae* cells were grown overnight in motility medium (MM) [36] at 25 °C with shaking. Cells were pelleted by centrifugation at 16,800×*g* for 15 min, and the culture supernatant (spent medium) was centrifuged for another 15 min to remove residual cells. For whole-cell samples, the cells were washed once with phosphate-buffered saline (PBS) consisting of 137 mM NaCl, 2.7 mM KCl, 10 mM Na_2_PO_4_, and 2 mM KH_2_PO_4_ (pH 7.4) and suspended in the original culture volume of PBS. For whole cells, 10 µg cell protein was loaded on gels. For secreted proteins, the amount of spent medium that contained 10 µg cell protein before cell removal was loaded. All samples were boiled in SDS-PAGE loading buffer for 7 min. Western blot analyses were performed as previously described [37] using antibodies against ChiA at 1:5000 dilution [13].

### Cloning, protein production, and purification of CusD_I_ and CusD_II_

The genes encoding CusD_I_ (Fjoh_4558, residues 33–526) and CusD_II_ (Fjoh_4561, residues 26–505) were PCR amplified from genomic DNA for ligation-independent cloning into the pETite N-His vector (Lucigen Madison, WI, USA) according to the manufacturer’s instructions. For crystallization, an N-terminal truncation of CusD_II_ (residues 35–505) was created and expressed similarly in the pETite N-His vector. For all constructs, the N-terminal primer encoded a TEV cleavage site immediately downstream of the complementary 18-bp overlap (encoding the His tag) to create a TEV-cleavable His-tagged protein. The resulting plasmids were transformed into Rosetta(DE3) pLysS cells, and the resulting cells were plated on LB agar containing kanamycin and incubated overnight at 37 °C. Colonies were used to inoculate 2 × 1 L of Terrific Broth media supplemented with kanamycin and chloramphenicol for protein expression. Cells were grown at 37 °C to an OD_600_ of ~0.6, IPTG (isopropyl-β-d-thiogalactopyranoside) was added to a final concentration of 0.5 mM, and the cells were grown at room temperature (20–23 °C) for an additional 16 h. Cells were then harvested by centrifugation and flash-frozen in liquid N_2_. Cells were lysed in His Buffer (25 mM NaH_2_PO_4_, 500 mM NaCl, 20 mM imidazole pH 7.4) via sonication and cell debris was removed by centrifugation at 30,000×*g* for 30 min. His-tagged proteins were purified using a 5-mL HiTrap metal affinity cartridge (GE Healthcare) according to the manufacturer’s instructions. The cell lysate was applied to the column in His Buffer (25 mM NaH_2_PO_4_, 500 mM NaCl, 20 mM imidazole pH 7.4) and proteins were eluted with an imidazole (20–300 mM) gradient. The His tag was removed by incubation with TEV protease (1:100 molar ratio relative to protein) at room temperature for 2 h, then overnight at 4 °C while dialyzing against His Buffer. The cleaved protein was re-purified on the 5-ml Ni column to remove undigested target protein, the cleaved His tag, and His-tagged TEV protease. Purified proteins were dialyzed against 20 mM HEPES and 100 mM NaCl (pH 7.0), and concentrated using Vivaspin 15 (10 kDa cutoff) centrifugal concentrators (Vivaproducts, Inc.), prior to crystallization.

### Cloning, protein production, and purification of the ChiUL enzymes

The enzyme-encoding genes of the ChiUL were optimized for expression in *E. coli* and synthesized by Life Technologies (Carlsbad, USA). The genes, without signal peptide-encoding parts, were amplified by PCR, using the primers listed in Table S4 (Additional file 1), and ligated into pNIC-CH expression vectors by ligation-independent cloning. The resulting plasmids were used to transform BL21(DE3) cells. Overnight cultures were used to inoculate 0.5–1 L of Terrific Broth media supplemented with kanamycin. Cells were grown at 37 °C to an OD_600_ of ~0.5, IPTG was added (0.2 mM final concentration), and protein production continued at lower temperatures (ChiA_F, ChiA_N, and ChiA_C at 16 °C for 3 days, ChiA_M, ChiB, and GH20 at 25 °C for 2 days). The harvested cells were resuspended and lysed by sonication in 25 mM Tris, 500 mM NaCl, and 20 mM imidazole (pH 8) and centrifuged (75,000×*g*, 40 min) to remove insoluble matter. His-tagged enzymes were purified by nickel affinity chromatography as described above (Tris buffer, pH 8.0, 20 mM–1 M imidazole gradient). Eluted proteins were washed and concentrated using Amicon 10 kDa cutoff spin filter membranes (Millipore), into 50 mM Tris, pH 7.5. For the enzyme ChiA_C, protein fractions were eluted into tubes containing an equal volume of 200 mM citrate (pH 5.0) to prevent precipitation (theoretical pI 7.1). ChiA_C was washed and concentrated using 50 mM citrate buffer (pH 5.0). All proteins yielded >100 mg purified protein per liter of culture. For ChiA_M, a second ion exchange purification on a HiTrap SP FF column (GE Lifesciences) was performed. The protein was loaded onto the column in 50 mM sodium acetate, pH 5.0, and eluted by a linear gradient toward 100% 50 mM sodium acetate and 1 M NaCl, pH 5.0.

### Protein crystallization and data collection

Crystals were obtained of CusD_I_ (residues 33–526) via hanging drop vapor diffusion at 20 °C by mixing the protein (12.2 mg/ml) in a 1:1 ratio with 150 mM Tris–HCl, pH 8.5, and 27% sokolan CP5. These crystals were briefly soaked in 10 mM acetylchitopentaose prior to freezing with a solution of 20% ethylene glycol, and 80% crystallization media. However, the ligand was not present in the electron density, and further analysis revealed that this is likely because of a crystal contact made by aromatic stacking of key Trp residues in the predicted glycan-binding site between the protein molecules, which precludes ligand binding.

Diffraction quality crystals of CusD_II_ (residues 35–505) were obtained from the PegRx2 crystallization screen (Hampton Research) at 20 °C by mixing the protein (18.1 mg/ml and 1 mM chitopentaose) 1:1 with crystallization media from condition 48 (3% Dextran sulfate sodium salt, 0.1 M Bicine, pH 8.5, 15% Poly(ethylene) glycol 20,000). For data collection, crystals were frozen by quickly transferring into cryoprotectant containing 80% mother liquor, 20% ethylene glycol, and 10 mM acetylchitopentaose, and then flash-frozen in liquid nitrogen. As with CusD_I_, the ligand was not visible in the electron density as critical protein–protein interactions driving crystal formation overlap with the predicted glycan-binding site.

X-ray data for the CusD_I_ and CusD_II_ protein crystals were collected at the Life Sciences Collaborative Access Team (LS-CAT) ID-F and ID-G beamlines at the Advanced Photon Source at Argonne National Labs, Argonne, IL. X-ray data from CusD_I_ were processed with HKL2000 and scaled with SCALEPACK [38], while X-ray data of CusD_II_ were processed in Xia2. Both protein structures were determined via molecular replacement in Phaser [39] within the Phenix software package [40, 41]. The structure of a SusD homologue, BVU_2203, from *Bacteroides vulgatus* (PDB 4F7A) was used as a search model to determine the structure of CusD_I_, and a polypeptide chain of the refined model was used as the search model to determine the structure of CusD_II_. Both protein models were automatically built with the Autobuild subroutine of Phenix, followed by successive rounds of manual model building in Coot [42] and refinement in Phenix. Data collection and refinement statistics are presented in Table S1 (Additional file 1).

### Isothermal titration calorimetry

ITC measurements for CusD_I_ and CusD_II_ were performed on a low-volume (250 µL sample cell) TA instruments NanoITC. Proteins were dialyzed against 20 mM HEPES and 100 mM NaCl, pH 7.0, and acetyl-chitooligosaccharide solutions were prepared using the dialysis buffer. Protein (44.1–384 μM) was placed in the sample cell and the reference cell filled with deionized water. After equilibration of the temperature to 25 °C, a first injection of 0.75 μL was performed followed by 27 subsequent injections of 1.75 μL of 0.5–5 mM of the substrates listed in Table 1. The solution was stirred at 350 rpm and the resulting heat of reaction was measured. Data were analyzed by fitting to an independent binding site model using the NanoAnalyze software package (TA instruments). Due to the low affinity of CusD_I_ for GlcNAc, the n value was constrained to 1 during curve fitting to obtain the overall affinity of the protein for this ligand.

### Activity assays

All enzymatic reactions of the ChiUL enzymes were performed at 25 °C. For reactions on chitooligosaccharides (ChiOs) and chitin, the reactions were stopped by the addition of an equal volume of 50 mM H_2_SO_4_. Product analysis was performed using high-performance liquid chromatography (HPLC) using an RSLC system (Dionex) equipped with a Rezex RFQ-Fast Acid H^+^ (100 × 7.8 mm) column (Phenomenex, Torrance, CA, USA) operating at 85 °C. 8-µl samples were injected, and solutes eluted by isocratic flow of 5 mM H_2_SO_4_ using a flow rate of 1 ml/min. ChiOs and GlcNac were monitored at *λ*_210_ and quantified using standard curves of commercially available compounds. The pH optima of the enzymes were determined using chitotetraose as the substrate for ChiA_N, ChiA_C, and ChiB, and chitobiose for the GH20 enzyme. Various buffers (50 mM) in the pH range from 2.5 to 9.0 were used (Additional file 1: Figure S7): sodium citrate (pH 2.5–6.5), MES (2-(*N*-morpholino)ethanesulfonic acid; pH 5.1–7.0), Bis–Tris (pH 6.0–7.0), sodium phosphate (pH 6.5–8.0), and Tris (pH 7.5–9.0). To determine the kinetic parameters for the ChiO-active enzymes, reactions were set up using increasing concentrations of ChiO substrates in 50 mM buffer (at optimal pH) and the following enzyme concentrations: 1 nM ChiA_N for chitotetra-hexaose, 2 µM for chitotriose; 20 nM ChiA_C for chitotetra-hexaose, 0.5 µM for chitotriose; 1 nM GH20 for all substrates; and 10 nM ChiB for chitotetra-hexaose, 1 µM for chitotriose. Curve fitting was performed using Origin 8 (OriginLab), using the Michaelis–Menten or substrate inhibition (*v*_*o*_ = *V*_max_*[*S*]/(*K*_m_ + [*S*] × (1 + [*S*]/*K*_*i*_))) equations. Time-course studies were performed to analyze the action of the enzymes on crystalline chitin polysaccharides. 500-µl reactions (5 g/l chitin, 50 mM buffer) in 2-ml round-bottom tubes were incubated in Eppendorf Thermomixers, with shaking at 1000 rpm to prevent settling of substrate particles. Enzyme concentrations were as follows: ChiA_F 1 µM, ChiA_N, ChiA_C, and ChiB 10 µM, respectively. 5 µM of ChiA_N and ChiA_C were used in the synergy reaction. 10-µl aliquots were taken out at intervals, and the reactions were stopped by the addition of 10 µl H_2_SO_4_, followed by dilution by adding 180 µl H_2_O. After vacuum filtration using 96-well filter plates operated by a vacuum manifold (Millipore), the products were analyzed by HPLC as described above.

### Binding of CusD_I_, CusD_II_, and ChiA_M to insoluble substrates

100 µl of 2 mg/ml CusD protein was mixed with 100 µl 4% (w/v) Avicel, starch amylopectin, or colloidal chitin (pre-washed three times with an excess of binding buffer, 20 mM HEPES, 100 mM NaCl, pH 7.5), respectively, and incubated at 37 °C for 30 min. The mixtures were then centrifuged at 3000×*g* for 2 min, and the supernatants were removed as the unbound protein fractions. The polysaccharide pellets were washed with 200 µl of binding buffer and centrifuged again. All of the supernatant was removed, and the pellet was resuspended in 50 µl of SDS-PAGE loading buffer, heated at 100° C for 3 min, and then centrifuged. Proteins released from the polysaccharides represented the bound fraction of protein. SDS-PAGE was used to analyze the protein fractions, using CusD proteins incubated in binding buffer as control samples. None of the proteins precipitated as a result of the incubation at 37 °C.

ChiA_M (0.5 g/l) was mixed with insoluble polysaccharides (3 g/l; ground α- and β-chitin sieved to particle sizes of ≤0.85 mm, ball-milled α- and β-chitin, cellulose (Avicel, ball-milled Whatman filter paper sieved to particle sizes of ≤ 0.5 mm), ivory nut mannan, and barley mixed-linkage glucan [Megazyme]) in a total volume of 200 µl, containing 50 mM sodium phosphate (pH 6.5). The samples were incubated with shaking (1000 rpm) for 1 h at 25 °C. Supernatants containing the unbound protein (U) were collected following centrifugation for 2 min at 20,000×*g* at room temperature (22 °C). 200 µl of fresh buffer was then added to the samples followed by incubation as before, for 15 min. Proteins released in the wash step (W) were collected as described previously. 100 µl of 8 M urea was added to the polysaccharide pellets followed by incubation as before, for 10 min. Supernatants containing released/eluted proteins (E) were obtained after centrifugation as above. The collected fractions were analyzed by SDS-PAGE.

## Additional file

**Additional file 1: Tables S1–S3.** Strains, plasmids and primers used in the study, **Table S4.** X-ray data and refinement statistics. **Figure S1.** Chitin utilization of wild type *F. johnsoniae* and mutants, **Figure S2.** Western blot immunodetection of ChiA, **Figure S3.** Growth curves of cells carrying pYT353 (CusC_I_) on chitin and glucose, **Figure S4.** Growth curves of wild type cells and predicted cytoplasmic inner membrane sugar transporter mutants, **Figure S5.** Binding analysis of the CusD proteins to insoluble polysaccharides, **Figure S6.** Section of a structural alignment of *Serratia marcescens* chitinases (ChiA, B and C) and the ChiUL chitinases, **Figure S7.** pH profiles of the ChiUL enzymes.

## Abbreviations

PUL: polysaccharide utilization locus
ChiUL: chitin utilization locus
LPMO: lytic polysaccharide monooxygenase
Sus: starch utilization system
XyGUL: xyloglucan utilization locus
T9SS: type IX secretion system
TCS: two-component sensor/regulator system
Cus: chitin utilization system
GH: glycoside hydrolase family
ChiO: chitooligosaccharide
ITC: isothermal calorimetry
GlcNAc: N-acetylglucosamine
PDB: protein data bank
RMSD: root mean square deviation
CBM: carbohydrate-binding module
CYE: casitone yeast extract
IPTG: isopropyl-β-d-thiogalactopyranoside
HPLC: high-performance liquid chromatography

## References

[1] Vaaje-Kolstad G, Horn SJ, Sorlie M, Eijsink VGH (2013). The chitinolytic machinery of *Serratia marcescens*—a model system for enzymatic degradation of recalcitrant polysaccharides. FEBS J.

[2] Qin J, Li R, Raes J, Arumugam M, Burgdorf KS, Manichanh C, Nielsen T, Pons N, Levenez F, Yamada T (2010). A human gut microbial gene catalogue established by metagenomic sequencing. Nature.

[3] Edwards JE, McEwan NR, Travis AJ, Wallace RJ (2004). 16S rDNA library-based analysis of ruminal bacterial diversity. Anton Leeuw Int J G.

[4] Thomas F, Hehemann JH, Rebuffet E, Czjzek M, Michel G. Environmental and gut Bacteroidetes: the food connection. Front Microbiol. 2011;2. 10.3389/fmicb.2011.00093 PMC3129010 21747801

[5] Martens EC, Koropatkin NM, Smith TJ, Gordon JI (2009). Complex glycan catabolism by the Human gut microbiota: the Bacteroidetes Sus-like paradigm. J Biol Chem.

[6] Terrapon N, Lombard V, Gilbert HJ, Henrissat B (2015). Automatic prediction of polysaccharide utilization loci in Bacteroidetes species. Bioinformatics.

[7] Larsbrink J, Rogers TE, Hemsworth GR, McKee LS, Tauzin AS, Spadiut O, Klinter S, Pudlo NA, Urs K, Koropatkin NM (2014). A discrete genetic locus confers xyloglucan metabolism in select human gut Bacteroidetes. Nature.

[8] Cuskin F, Lowe EC, Temple MJ, Zhu YP, Cameron EA, Pudlo NA, Porter NT, Urs K, Thompson AJ, Cartmell A (2015). Human gut Bacteroidetes can utilize yeast mannan through a selfish mechanism. Nature.

[9] Rosewarne CP, Pope PB, Cheung JL, Morrison M (2014). Analysis of the bovine rumen microbiome reveals a diversity of Sus-like polysaccharide utilization loci from the bacterial phylum Bacteroidetes. J Ind Microbiol Biotechnol.

[10] Mackenzie AK, Naas AE, Kracun SK, Schuckel J, Fangel JU, Agger JW, Willats WGT, Eijsink VGH, Pope PB (2015). A polysaccharide utilization locus from an uncultured Bacteroidetes phylotype suggests ecological adaptation and substrate versatility. Appl Environ Microbiol.

[11] Naas AE, Mackenzie AK, Mravec J, Schuckel J, Willats WGT, Eijsink VGH, Pope PB (2014). Do rumen Bacteroidetes utilize an alternative mechanism for cellulose degradation. mBio.

[12] McBride MJ, Xie G, Martens EC, Lapidus A, Henrissat B, Rhodes RG, Goltsman E, Wang W, Xu J, Hunnicutt DW (2009). Novel features of the polysaccharide-digesting gliding bacterium *Flavobacterium johnsoniae* as revealed by genome sequence analysis. Appl Environ Microbiol.

[13] Kharade SS, McBride MJ (2014). Flavobacterium johnsoniae chitinase ChiA is required for chitin utilization and is secreted by the type IX secretion system. J Bacteriol.

[14] McBride MJ, Zhu Y (2013). Gliding motility and Por secretion system genes are widespread among members of the phylum Bacteroidetes. J Bacteriol.

[15] Shipman JA, Cho KH, Siegel HA, Salyers AA (1999). Physiological characterization of SusG, an outer membrane protein essential for starch utilization by *Bacteroides thetaiotaomicron*. J Bacteriol.

[16] Petersen TN, Brunak S, von Heijne G, Nielsen H (2011). SignalP 4.0: discriminating signal peptides from transmembrane regions. Nat Methods.

[17] D’Elia JN, Salyers AA (1996). Effect of regulatory protein levels on utilization of starch by *Bacteroides thetaiotaomicron*. J Bacteriol.

[18] Reeves AR, D’Elia JN, Frias J, Salyers AA (1996). A *Bacteroides thetaiotaomicron* outer membrane protein that is essential for utilization of maltooligosaccharides and starch. J Bacteriol.

[19] Bolam DN, Koropatkin NM (2012). Glycan recognition by the Bacteroidetes Sus-like systems. Curr Opin Struct Biol.

[20] Nakamura T, Mine S, Hagihara Y, Ishikawa K, Ikegami T, Uegaki K (2008). Tertiary structure and carbohydrate recognition by the chitin-binding domain of a hyperthermophilic chitinase from *Pyrococcus furiosus*. J Mol Biol.

[21] Hanazono Y, Takeda K, Niwa S, Hibi M, Takahashi N, Kanai T, Atomi H, Miki K (2016). Crystal structures of chitin binding domains of chitinase from *Thermococcus kodakarensis* KOD1. FEBS Lett.

[22] Horn SJ, Sorlie M, Vaaje-Kolstad G, Norberg AL, Synstad B, Varum KM, Eijsink VGH (2006). Comparative studies of chitinases A, B and C from *Serratia marcescens*. Biocatal Biotransfor.

[23] Brurberg MB, Nes IF, Eijsink VG (1996). Comparative studies of chitinases A and B from *Serratia marcescens*. Microbiology..

[24] Brunecky R, Alahuhta M, Xu Q, Donohoe BS, Crowley MF, Kataeva IA, Yang SJ, Resch MG, Adams MWW, Lunin VV (2013). Revealing nature’s cellulase diversity: the digestion mechanism of *Caldicellulosiruptor bescii* CelA. Science.

[25] Blumer-Schuette SE, Giannone RJ, Zurawski JV, Ozdemir I, Ma Q, Yin Y, Xu Y, Kataeva I, Poole FL, Adams MW (2012). *Caldicellulosiruptor* core and pangenomes reveal determinants for noncellulosomal thermophilic deconstruction of plant biomass. J Bacteriol.

[26] Stern J, Kahn A, Vazana Y, Shamshoum M, Morais S, Lamed R, Bayer EA (2015). Significance of relative position of cellulases in designer cellulosomes for optimized cellulolysis. PLoS ONE.

[27] Vanfossen AL, Verhaart MR, Kengen SM, Kelly RM (2009). Carbohydrate utilization patterns for the extremely thermophilic bacterium *Caldicellulosiruptor saccharolyticus* reveal broad growth substrate preferences. Appl Environ Microbiol.

[28] Reichenbach H, Balows A, Truper H, Dworkin M, Harder W, Schleifer K (1992). The genus *Lysobacter*. The Prokaryotes.

[29] Chang LYE, Pate JL, Betzig RJ (1984). Isolation and characterization of nonspreading mutants of the gliding bacterium *Cytophaga jonhsonae*. J Bacteriol.

[30] Rhodes RG, Pucker HG, McBride MJ (2011). Development and use of a gene deletion strategy for *Flavobacterium johnsoniae* to identify the redundant gliding motility genes *remF*, *remG*, *remH*, and *remI*. J Bacteriol.

[31] McBride MJ, Kempf MJ (1996). Development of techniques for the genetic manipulation of the gliding bacterium *Cytophaga johnsonae*. J Bacteriol.

[32] Sambrook J, Fritsch EF, Maniatis T (1989). Molecular cloning: a laboratory manual.

[33] Hunnicutt DW, McBride MJ (2000). Cloning and characterization of the *Flavobacterium johnsoniae* gliding motility genes, *gldB* and *gldC*. J Bacteriol.

[34] Stanier RY (1942). The *Cytophaga* group: a contribution to the biology of myxobacteria. Bacteriol Rev.

[35] Zhu Y, Kwiatkowski KJ, Yang T, Kharade SS, Bahr CM, Koropatkin NM, Liu W, McBride MJ. Outer membrane proteins related to SusC and SusD are not required for *Cytophaga hutchinsonii* cellulose utilization. Appl Microbiol Biotechnol. 2015. 10.1007/s00253-015-6555-8 25846333

[36] Liu J, McBride MJ, Subramaniam S (2007). Cell surface filaments of the gliding bacterium *Flavobacterium johnsoniae* revealed by cryo-electron tomography. J Bacteriol.

[37] Rhodes RG, Samarasam MN, Shrivastava A, van Baaren JM, Pochiraju S, Bollampalli S, McBride MJ (2010). *Flavobacterium johnsoniae gldN* and *gldO* are partially redundant genes required for gliding motility and surface localization of SprB. J Bacteriol.

[38] Otwinowski Z, Minor W (1997). Processing of X-ray diffraction data collected in oscillation mode. Methods Enzymol..

[39] McCoy AJ, Grosse-Kunstleve RW, Adams PD, Winn MD, Storoni LC, Read RJ (2007). Phaser crystallographic software. J Appl Crystallogr.

[40] Adams PD, Grosse-Kunstleve RW, Hung LW, Ioerger TR, McCoy AJ, Moriarty NW, Read RJ, Sacchettini JC, Sauter NK, Terwilliger TC (2002). PHENIX: building new software for automated crystallographic structure determination. Acta Crystallogr D Biol Crystallogr.

[41] Zwart PH, Afonine PV, Grosse-Kunstleve RW, Hung LW, Ioerger TR, McCoy AJ, McKee E, Moriarty NW, Read RJ, Sacchettini JC (2008). Automated structure solution with the PHENIX suite. Methods Mol Biol.

[42] Emsley P, Cowtan K (2004). Coot: model-building tools for molecular graphics. Acta Crystallogr D Biol Crystallogr.

