# Supplementary material for: A polysaccharide utilization locus from Flavobacterium johnsoniae enables conversion of recalcitrant chitin
Source: Biotechnol Biofuels. 2016 Nov 28;9:260. doi: 10.1186/s13068-016-0674-z (PMC5127042; doi:10.1186/s13068-016-0674-z)
Supplement: Supplementary file 1 — Additional file 1: Tables S1–S3. Strains, plasmids and primers used in the study, Table S4. X-ray data and refinement statistics. Figure S1. Chitin utilization of wild type F. johnsoniae and mutants, Figure S2. Western blot immunodetection of ChiA, Figure S3. Growth curves of cells carrying pYT353 (CusCI) on chitin and glucose, Figure S4. Growth curves of wild type cells and predicted cytoplasmic inner membrane sugar transporter mutants, Figure S5. Binding analysis of the CusD proteins to insoluble polysaccharides, Figure S6. Section of a structural alignment of Serratia marcescens chitinases (ChiA, B and C) and the ChiUL chitinases, Figure S7. pH profiles of the ChiUL enzymes. [file 13068_2016_674_MOESM1_ESM.pdf]

Supplementary information

Tables

Table S1: Bacterial strains used in the study

Strain	Description*	Source
E. coli strains		
BL21(DE3)	Strain used for production of ChiUL enzymes	Invitrogen (Carlsbad, US)
DH5αMCR	Strain used for general cloning	Life Technologies (Grand Island, NY, USA)
HB101	Strain used for triparental conjugation	[1]
Rosetta(DE3) pLysS	Strain used for production of CusD proteins	EMD Millipore (Darmstadt, Germany)
F. johnsoniae strains		
UW101 (ATCC 17061^T)	Wild type	
CJ1808	*chiA* disruption mutant; (Em^r)	[2]
CJ1827	*rpsL2*; (Sm^r) "wild type" strains for construction of all deletion mutants	[3]
CJ2018	*rpsL2* Δ*cusD$_{II}$* (Fjoh_4561); (Sm^r)	This study
CJ2086	*rpsL2* Δ*cusC$_I$* (Fjoh_4559); (Sm^r)	This study
CJ2121	*rpsL2* Δ*cusD$_I$* (Fjoh_4558); (Sm^r)	This study
CJ2156	*rpsL2* Δ*cusD$_I$* Δ*cusD$_{II}$*; (Sm^r)	This study
CJ2340	*rpsL2* Δ*cusC$_{II}$* (Fjoh_4562); (Sm^r)	This study
CJ2349	*rpsL2* Δ(*cusC$_I$* - *cusD$_{II}$*); (Sm^r)	This study
CJ2350	*rpsL2* Δ*chiB* (Fjoh_4560); (Sm^r)	This study
CJ2453	*rpsL2* Δ(*cusS-cusR*); (Sm^r)	This study
CJ2456	*rpsL2* ΔFjoh_4556; (Sm^r)	This study
CJ2457	*rpsL2* ΔFjoh_4565; (Sm^r)	This study
CJ2463	*rpsL2* Δ*nagB* (Fjoh_4557); (Sm^r)	This study
CJ2590	*rpsL2* Δ*cusC$_I$* Δ*cusC$_{II}$*; (Sm^r)	This study
CJ2676	*rpsL2* Δ(*cusC$_{II}$* - *cusD$_{II}$*); (Sm^r)	This study
CJ2677	*rpsL2* Δ(*cusC$_I$* - *cusD$_I$*); (Sm^r)	This study
CJ2679	*rpsL2* Δ(*cusC$_I$* - *cusD$_I$*) Δ(*cusC$_{II}$* - *cusD$_{II}$*); (Sm^r)	This study

*Antibiotic resistance phenotypes: ampicillin, Ap^r; erythromycin, Em^r; kanamycin, Km^r; streptomycin, Sm^r; tetracycline, Tc^r. Unless indicated otherwise, the antibiotic resistance phenotypes are those expressed in *E. coli*. The antibiotic resistance phenotypes given in parentheses are those expressed in *F. johnsoniae* but not in *E. coli*

Table S2: Plasmids used in this study

Plasmid	Description*	Source
pCP23	*E. coli*/ *F. johnsoniae* shuttle vector; Ap^r (Tc^r)	[4]
pDE01	1.7 kbp region upstream of *nagB* amplified with primers 1626 and 1627 and cloned into XbaI and SalI sites of pRR51; Ap^r (Em^r)	This study
pDE02	Construct used to delete *nagB*; 1.7 kbp region downstream of *nagB* amplified with primers 1628 and 1629 and cloned into SalI and SphI sites of pDE01; Ap^r (Em^r)	This study
pETite N-His	Expression plasmid, including an N-terminal His$_6$-tag and TEV protease cleavage site; (Km^r)	Lucigen
pLW01	pCP23 carrying *cusD*$_I$; Ap^r (Tc^r)	This study
pNIC-CH	Expression plasmid, including a C-terminal His$_6$-tag; (Km^r)	Addgene
pRK2013	Helper plasmid for triparental conjugation; IncP Tra$^+$ Km^r	[5]

pRR51	*rpsL*-containing suicide vector for construction of deletions; Ap^r (Em^r)	[3]
pSSK05	pCP23 carrying *chiA*; Ap^r (Tc^r)	[2]
pSSK08	1.8 kbp region downstream of $cusD_{II}$ amplified with primers 1055 and 1056 and cloned into BamHI and SalI sites of pRR51; Ap^r (Em^r)	This study
pSSK09	Construct used to delete $cusD_{II}$; 1.3 kbp region upstream of $cusD_{II}$ amplified with primers 1057 and 1058 and cloned into SalI and SphI sites of pSSK08; Ap^r (Em^r)	This study
pSSK13	1.7 kbp region downstream of $cusD_I$ amplified with primers 1166 and 1167 and cloned into XbaI and SalI sites of pRR51; Ap^r (Em^r)	This study
pSSK15	1.5 kbp region downstream of $cusC_I$ amplified with primers 1170 and 1052 and cloned into XbaI and SalI sites of pRR51; Ap^r (Em^r)	This study
pSSK17	Construct used to delete $cusC_I$; 1.8 kbp region upstream of $cusC_I$ amplified with primers 1053 and 1054 and cloned into SalI and SphI sites of pSSK15; Ap^r (Em^r)	This study
pSSK18	Construct used to delete $cusD_I$; 1.7 kbp region upstream of $cusD_I$ amplified with primers 1168 and 1169 and cloned into SalI and SphI sites of pSSK13; Ap^r (Em^r)	This study
pSSK29	1.9 kbp region downstream of $cusC_{II}$ amplified with primers 1250 and 1251 and cloned into XbaI and SalI sites of pRR51; Ap^r (Em^r)	This study
pSSK33	Construct used to delete $cusC_{II}$; 1.8 kbp region upstream of $cusC_{II}$ amplified with primers 1252 and 1253 and cloned into SalI and SphI sites of pSSK29; Ap^r (Em^r)	This study
pSSK38	1.5 kbp region upstream of *chiB* amplified with primers 1423 and 1424 and cloned into BamHI and SalI sites of pRR51; Ap^r (Em^r)	This study
pSSK40	Construct used to delete *chiB*; 1.6 kbp region downstream of *chiB* amplified with primers 1425 and 1426 and cloned into SalI and SphI sites of pSSK38; Ap^r (Em^r)	This study
pSSK49	1,518-bp NheI-XhoI fragment spanning $cusD_I$ amplified with primer 1561 and 1562 and inserted into pET28a	This study
pSSK50	1,461-bp NheI-XhoI fragment spanning $cusD_{II}$ amplified with primer 1563 and 1564 and inserted into pET28a	This study
pYT248	2.1 kbp region upstream of Fjoh_4556 amplified with primers 1622 and 1623 and cloned into BamHI and SalI sites of pRR51; Ap^r (Em^r)	This study
pYT249	2.0 kbp region upstream of *cusS* amplified with primers 1630 and 1631 and cloned into BamHI and SalI sites of pRR51; Ap^r (Em^r)	This study
pYT250	1.9 kbp region upstream of Fjoh_4565 amplified with primers 1634 and 1635 and cloned into BamHI and SalI sites of pRR51; Ap^r (Em^r)	This study
pYT255	Construct used to delete Fjoh_4556; 2.0 kbp region downstream of Fjoh_4556 amplified with primers 1624 and 1625 and cloned into SalI and SphI sites of pYT248; Ap^r (Em^r)	This study
pYT256	Construct used to delete *cusS* - *cusR*; 1.8 kbp region downstream of *cusR* amplified with primers 1632 and 1633 and cloned into SalI and SphI sites of pYT249; Ap^r (Em^r)	This study
pYT257	Construct used to delete Fjoh_4565; 1.8 kbp region downstream of Fjoh_4565 amplified with primers 1636 and 1637 and cloned into SalI and SphI sites of pYT250; Ap^r (Em^r)	This study
pYT259	pCP23 carrying *cusS* -*cusR*; Ap^r (Tc^r)	This study
pYT260	pCP23 carrying Fjoh_4565; Ap^r (Tc^r)	This study
pYT351	pCP23 carrying $cusC_I$ - $cusD_I$; Ap^r (Tc^r)	This study
pYT352	pCP23 carrying $cusC_{II}$ - $cusD_{II}$; Ap^r (Tc^r)	This study
pYT353	pCP23 carrying $cusC_I$; Ap^r (Tc^r)	This study

*Antibiotic resistance phenotypes: ampicillin, Ap^r; erythromycin, Em^r; kanamycin, Km^r; streptomycin, Sm^r; tetracycline, Tc^r. Unless indicated otherwise, the antibiotic resistance phenotypes are those expressed in *E. coli*. The antibiotic resistance phenotypes given in parentheses are those expressed in *F. johnsoniae* but not in *E. coli.*

Table S3: Primers used in this study

Primers	Sequence and Description
ChiA_F_F	5' TTAAGAAGGAGATATACTATGAAAGTTGTTGGTTATTATGCCCAGTGG 3'; forward primer used to clone ChiA_F (Fjoh_4555); LIC overhang underlined
ChiA_F_R	GTAATAGCAGTGAACTGAGCAAAGCAGCGCACCATCATCACCACCATT 3'; reverse primer used to clone ChiA_F (Fjoh_4555); LIC overhang underlined
ChiA_N_F	5' TTAAGAAGGAGATATACTATGAAAGTTGTTGGTTATTATGCCCAGTGG 3'; forward primer used to clone ChiA_N (Fjoh_4555); LIC overhang underlined
ChiA_N_R	GATACCAGCTTTGGTAGCGTGGTTGCGCACCATCATCACCACCATT 3'; reverse primer used to clone ChiA_N (Fjoh_4555); LIC overhang underlined
ChiA_M_F	5' TTAAGAAGGAGATATACTATGGGTAGCGTGGTTCCGGGTAC 3'; forward primer used to clone ChiA_M (Fjoh_4555); LIC overhang underlined
ChiA_M_R	CAACCGTGAGCACCAATCGTTTTATTGCGCACCATCATCACCACCATT 3'; reverse primer used to clone ChiA_M (Fjoh_4555); LIC overhang underlined
ChiA_C_F	5' TTAAGAAGGAGATATACTATGGTTGTGATTAAAGCAACCGACAATAAAAG 3'; forward primer used to clone ChiA_C (Fjoh_4555); LIC overhang underlined
ChiA_C_R	GTAATAGCAGTGAACTGAGCAAAGCAGCGCACCATCATCACCACCATT 3'; reverse primer used to clone ChiA_C (Fjoh_4555); LIC overhang underlined
GH20_F	5' TTAAGAAGGAGATATACTATGCAGATGCAGAAAGAACAGCTGAATC 3'; forward primer used to clone GH20 (Fjoh_4556); LIC overhang underlined
GH20_R	CGTTTATGAAAGCCTGAAAAAACTGATTGCGCACCATCATCACCACCATT 3'; reverse primer used to clone GH20 (Fjoh_4556); LIC overhang underlined
ChiB_F	5' TTAAGAAGGAGATATACTATGACCAGCGAAAAAGAAAATAACCCGGAAG 3'; forward primer used to clone ChiB (Fjoh_4560); LIC overhang underlined
ChiB_R	CAAAACCAGCGGTATGTGTGGCAACGCGCACCATCATCACCACCATT 3'; reverse primer used to clone ChiB (Fjoh_4560); LIC overhang underlined
CusD$_{I}$_F	5’ CATCATCACCACCATCACGAGAACCTGTACTTCCAGGGCAATGGTATTACACTTCCTGATTTTG 3’; forward primer used to clone CusD$_{I}$ (Fjoh_4558); LIC overhang and TEV site underlined
CusD$_{I}$_R	5’ GTGGCGGCCGCTCTATTAGAAATTCGGAGCATTTACATCC 3’; reverse primer used to clone CusD$_{I}$ (Fjoh_4558); LIC overhang underlined
CusD$_{II}$_F1	5’ CATCATCACCACCATCACGAGAACCTGTACTTCCAGGGCCTGATAAAGGATCCGGTTGCG 3’; forward primer used to clone CusD$_{II}$ (Fjoh_4561) residues 26-505; LIC overhang and TEV site underlined
CusD$_{II}$_F2	5’ CATCATCACCACCATCACGAGAACCTGTACTTCCAGGGCCCAAATCCTG CAGGTCAGCTTAC 3’; forward primer used to clone CusD$_{II}$ (Fjoh_4561) residues 35-505; LIC overhang and TEV site underlined
CusD$_{II}$_R	5’ GTGGCGGCCGCTCTATTAGTTTACATCCCACCAAACCTTAC 3’; reverse primer used to clone CusD$_{II}$ (Fjoh_4561); LIC overhang underlined
1052	5' GCTAGGTCGACACAGGTGATGCAAGAAATGCAGGC 3'; reverse primer used in construction of pSSK15; SalI site underlined
1053	5' GCTAGGTCGACTTTTACCTGTGCAAGCGAAACCTG 3'; forward primer used in construction of pSSK17; SalI site underlined
1054	5' GCTAGGCATGCGCTCCTGCAAGTCAGGCAAGTATT 3'; reverse primer used in construction of pSSK17; SphI site underlined
1055	5' GCTAGGGATCCTTTTACCTGTGCAAGCGAAACCTG 3'; forward primer used in construction of pSSK08; BamHI site underlined
1056	5' GCTAGGTCGACGCTCCTGCAAGTCAGGCAAGTATT 3'; reverse primer used in construction of pSSK08; SalI site underlined
1057	5' GCTAGGTCGACTGTAAGCTGACCTGCAGGATTTGG 3'; forward primer used in construction of pSSK09; SalI site underlined
1058	5' GCTAGGCATGCAATGCACCGGGAGCTTACAAGAAC 3'; reverse primer used in construction of pSSK09; SphI site underlined
1166	5' GCTAGTCTAGATACAATTTCGATATCCTCCTGCCC 3'; forward primer used in construction of pSSK13; XbaI site underlined
1167	5' GCTAGGTCGACGGAGTTTCTAAATTGGGCGGACCA 3'; reverse primer used in construction of pSSK13; SalI site underlined
1168	5' GCTAGGTCGACGGCGAGTAACAAAGTACAAATAGTTGCTTT 3'; forward primer used in construction of pSSK18; SalI site underlined
1169	5' GCTAGGCATGCTGGTTGTCGATTGCTTCTAGATACAGTTAT 3'; reverse primer used in construction of pSSK18; SphI site underlined
1170	5' GCTAGTCTAGACTGAGCAGTACCGCCCATATTCCA 3'; forward primer used in construction of pSSK15; XbaI site underlined
1250	5' GCTAGGTCGACGTAGCAAATGGAGTTGTTAATACAGGA 3'; reverse primer used in construction of pSSK29; SalI site underlined

1251	5' GCTAGTCTAGAAGTTTACATCCCACCAAACCTTACCAG3'; forward primer used in construction of pSSK29; XbaI site underlined
1252	5' GCTAGGCATGCCTGCTGCAAATTCTCTAAAAGC 3'; reverse primer used in construction of pSSK33; SphI site underlined
1253	5' GCTAGGTCGACAGCAGATGCCTGAATCGTATACATACC 3'; forward primer used in construction of pSSK33; SalI site underlined
1423	5' GCTAGGGATCCTTTACTCAATTATGTATGTCTGGAGAC 3'; forward primer used in construction of pSSK38; BamHI site underlined
1424	5' GCTAGGTCGACAAGGATGCCTAATAAGGCTTTATTTTT 3'; reverse primer used in construction of pSSK38; SalI site underlined
1425	5' GCTAGGTCGACTACAAGACTTCAGGCATGTGCGGT 3'; forward primer used in construction of pSSK40; SalI site underlined
1426	5' GCTAGGCATGCTATAATAATTAAAGTCATTTCCTCTTG 3'; reverse primer used in construction of pSSK40; SphI site underlined
1512	5' GCTAGGGTACCGGAACTGGCTCAGGATTCTT 3'; forward primer used in construction of pYT351 and pYT353; KpnI site underlined
1515	5' GCTAGGCATGCCTGCTTGTACCATTTGCTAACC 3'; reverse primer used in construction of pLW01 and pYT351; SphI site underlined
1561	5' GCTAGGCTAGCACAGATAATTTTGAAGACATTAATACT 3'; forward primer used in construction of pSSK49; NheI site underlined
1562	5' GCTAGCTCGAGTTAGAAATTCGGAGCATTTACATCCCA 3'; reverse primer used in construction of pSSK49; XhoI site underlined
1563	5' GCTAGGCTAGCACAGAAAATTTTGACGAACTGATAAAG 3'; forward primer used in construction of pSSK50; NheI site underlined
1564	5' GCTAGCTCGAGTTAGTTTACATCCCACCAAACCTTACC 3'; reverse primer used in construction of pSSK50; XhoI site underlined
1622	5' GCTAGGGATCCGAAGCTTCGGTAATTGTAGCACAGG 3'; forward primer used in construction of pYT248; BamHI site underlined
1623	5' GCTAGGTCGACTTCTTTTTGCATTTGAGCATTTGC 3'; reverse primer used in construction of pYT248; SalI site underlined
1624	5' GCTAGGTCGACAAAGATCATGCTGACGTAGAGTTG 3'; forward primer used in construction of pYT255; SalI site underlined
1625	5' GCTAGGCATGCGCATTTCGTAATTCAACTGTAACAT 3'; reverse primer used in construction of pYT255; SphI site underlined
1626	5' GCTAGTCTAGAGTACTTTGTTACTCGCCGCTGTG 3'; forward primer used in construction of pDE01; XbaI site underlined
1627	5' GCTAGGTCGACGGCGCTCTTATAACTGATATCAGG 3'; reverse primer used in construction of pDE01; SalI site underlined
1628	5' GCTAGGTCGACAGAGAATTCTGGGTTAGAGCCGAAG 3'; forward primer used in construction of pDE02; SalI site underlined
1629	5' GCTAGGCATGCGGCAAGTTCTGTCCACATTGTAGC 3'; reverse primer used in construction of pDE02; SphI site underlined
1630	5' GCTAGGGATCCTCCTGTCTTCTAAAATCTCTGCGG 3'; forward primer used in construction of pYT249; BamHI site underlined
1631	5' GCTAGGTCGACGTAGCTTCCCCAAAACCATATATGA 3'; reverse primer used in construction of pYT249; SalI site underlined
1632	5' GCTAGGTCGACCAAATTCAGTCGTACACGATTCCTA 3'; forward primer used in construction of pYT256; SalI site underlined
1633	5' GCTAGGCATGCCGCTAAGTAAAAGGGCCAAAGGT 3'; reverse primer used in construction of pYT256 and pYT260; SphI site underlined
1634	5' GCTAGGGATCCTTTACTCTCAACTTTTTACGATGGG 3'; forward primer used in construction of pYT250; BamHI site underlined
1635	5' GCTAGGTCGACAAACTGTCCCCATTTGGTTTGAAC 3'; reverse primer used in construction of pYT250; SalI site underlined
1636	5' GCTAGGTCGACTTGAAAAAACAAGGAATCAGCCAC 3'; forward primer used in construction of pYT257; SalI site underlined
1637	5' GCTAGGCATGCCGGATAAGGTTTACGAAAAAATGCT 3'; reverse primer used in construction of pYT257; SphI site underlined
1650	5' GCTAGGGATCCTTGAATAATAATACTTACTCCCGGC 3'; forward primer used in construction of pYT259; BamHI site underlined
1651	5' GCTAGGCATGCCCAACTACCTTATTTATTCAACAATGTG 3'; reverse primer used in construction of pYT259; SphI site underlined
1652	5' GCTAGGGATCCCGATACCACAAAAGGCGATTCAC 3'; forward primer used in construction of pYT260; BamHI site underlined

1871	5' GCTAGGGTACCCTACACAACTGTTGGAGGAAGA 3'; forward primer used in construction of pLW01; KpnI site underlined
1955	5' GCTAGGGTACCATCATTAAAGTAAGCACCCCATCG 3'; forward primer used in construction of pYT352; KpnI site underlined
1956	5' GCTAGGCATGCCCTTATCAGGCATAACTATATTTTAGG 3'; reverse primer used in construction of pYT352; SphI site underlined
1957	5' GCTAGGCATGCCGGCGAGTAACAAAGTACAAATAG 3'; reverse primer used in construction of pYT353; SphI site underlined

Table S4: X-ray Data Collection and Refinement Statistics

	$CusD_I$ (Fjoh_4558)	$CusD_{II}$ (Fjoh_4561)
PDB	5J90	5J5U
Resolution range (Å)	36.57 – 1.393	44.49 – 2.3
Space group	P 21 21 21	P 1
Unit cell (Å)	79.33, 112.14, 121.57<br>α=β=γ=90°	82.71 82.89 93.91<br>α=66.4°, β=78.3° γ=67.6°
Total reflections	1693675 (129785)	357070 (35307)
Unique reflections	215491 (21033)	91093 (9016)
Multiplicity	7.9 (6.2)	3.9 (3.9)
Completeness (%)	99.81 (98.30)	97.26 (96.68)
Mean I/sigma(I)	26.56 (4.63)	7.88 (4.14)
Wilson B-factor	11.53	22.6
R_{merge}	0.05585 (0.3396)	0.123 (0.3061)
R_{meas}	0.05968	0.1424
CC1/2	0.998 (0.941)	0.986 (0.909)
CC*	0.999 (0.985)	0.996 (0.976)
R_{work}	0.1503 (0.1911)	0.2139 (0.2566)
R_{free}	0.1673 (0.2213)	0.2626 (0.3454)
Number of non-hydrogen atoms	9149	14777
macromolecules	7591	14157
ligand (ethylene glycol)	44	0
water	1514	620
Protein residues	964	1806
RMS bonds (Å)	0.006	0.009
RMS angles (°)	1.08	1.14
Ramachandran favored (%)	97	97
Ramachandran allowed (%)	3	3
Ramachandran outliers (%)	0	0
Clashscore	0.8	3.1
Average B-factor	15.7	26
macromolecules	13.5	26
ligands (ethylene glycol)	24.1	
solvent	26.4	25.3

Values in parenthesis are for the highest resolution shell

Figures

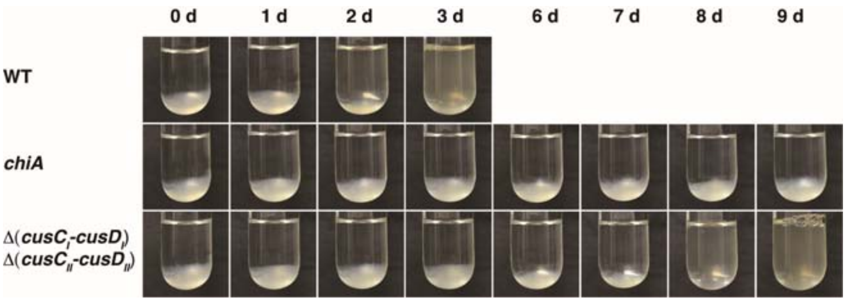

Figure S1: Chitin utilization of wild type F. johnsoniae and mutants. Chitin utilization was observed as the disappearance of insoluble chitin at the bottom of the test tubes, and growth was observed as increased turbidity throughout the culture. The chiA mutant was used as a negative control that exhibited no growth and no chitin conversion. Cells (0.02 ml, OD_{600}=1.0) were introduced into 10 ml of Stanier medium supplemented with 0.05% chitin in 150 mm by 25 mm test tubes and incubated at 25°C. Growth experiments were performed in duplicate (with the same results) and one set of data is shown.

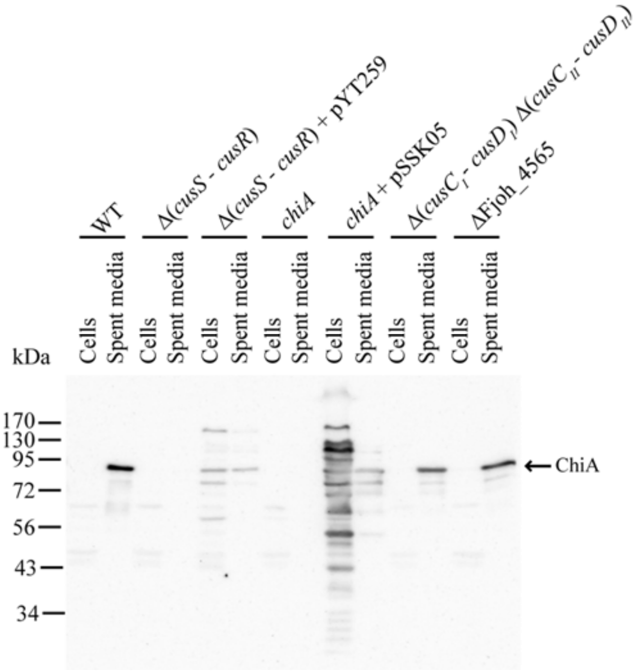

Figure S2: Western blot immunodetection of ChiA. F. johnsoniae wild type and mutant cells were grown overnight in motility medium at 25°C with shaking. Cells and supernatants (spent media) were separated by centrifugation. For whole cells, 10 µg cell protein was loaded on gels. For secreted proteins, the amount of spent medium that contained 10 µg cell protein before cell removal was loaded. Western blot analyses were performed using antibodies against ChiA at 1: 5,000 dilution. pYT259 expresses the two component signal transduction proteins CusS and CusR, and pSSK05 expresses ChiA. The observation of ChiA as a ~95 kDa band (the expected size is 169 kDa) is consistent with previous studies, which used the same antibodies as in

the current study, raised against a part of the N-terminal region of the protein [2]. The ChiUL does not encode proteases, and we assume that ChiA_F is proteolytically cleaved by secreted proteases during growth, similar to how the well-studied Serratia marcescens chitinases are susceptible to cleavage by endogenously produced proteases, whereby chitinolytic activity is decreased [6, 7]. ChiA_F does not exhibit detectable auto-lytic activities (no degradation observed by SDS-PAGE after incubation at 22°C for two days in 50 mM sodium phosphate, pH 6.5). Given that ChiA_F is dramatically more active on chitin as full-length protein compared to the individual GH18 domains (ChiA_N and ChiA_C, singly or combined), it is likely that full-length ChiA is responsible for chitin utilization by F. johnsoniae. Further, the strong affinity of the middle region of ChiA (ChiA_M, Figure 6) to chitin crystals, suggests that ChiA_F is in intimate contact with the substrate during degradation. Note that only soluble fractions were analyzed in the Western blot immunodetection experiments. It is possible that ChiA_F, protected from proteases while bound to its insoluble substrate, was not detected for this reason.

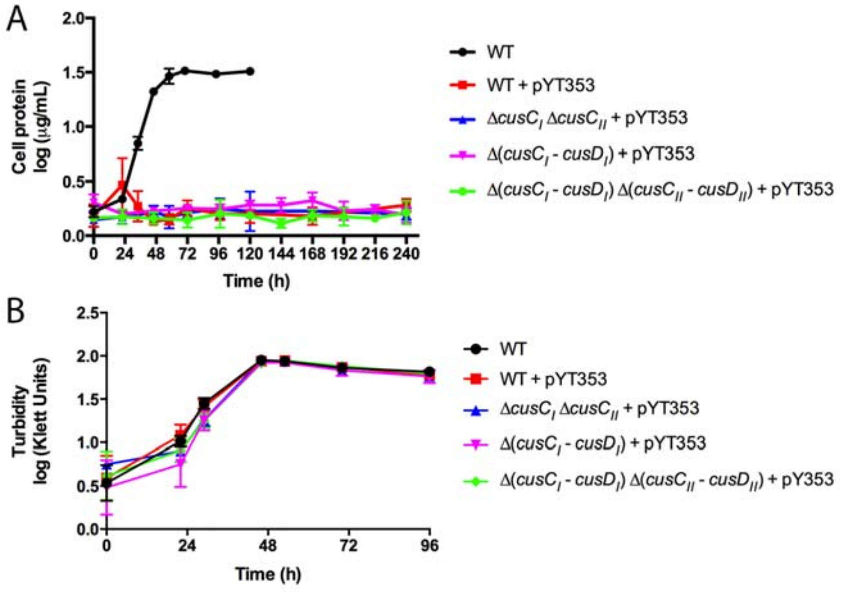

Figure S3: Growth curves of cells carrying pYT353 (cusC$_I$) on chitin (A) and glucose (B). For growth on chitin, cells (0.1 ml, OD_{600}=1.0) were introduced into 50 ml of Stanier medium supplemented with 0.05% chitin in 250-ml flasks, and incubated with shaking at 25°C. Growth presented as log (µg cell protein/ml). For growth on glucose, cells (0.1 ml, OD_{600}=1.0) were introduced into 50 ml of Stanier medium supplemented with 0.1% glucose in 250-ml side-arm flasks and incubated with shaking at 25°C. Growth presented as log (Klett Units). Growth curves were performed in triplicate and error bars indicate standard deviations. pYT353 is derived from pCP23, which has a copy number of approximately 10 in F. johnsoniae.

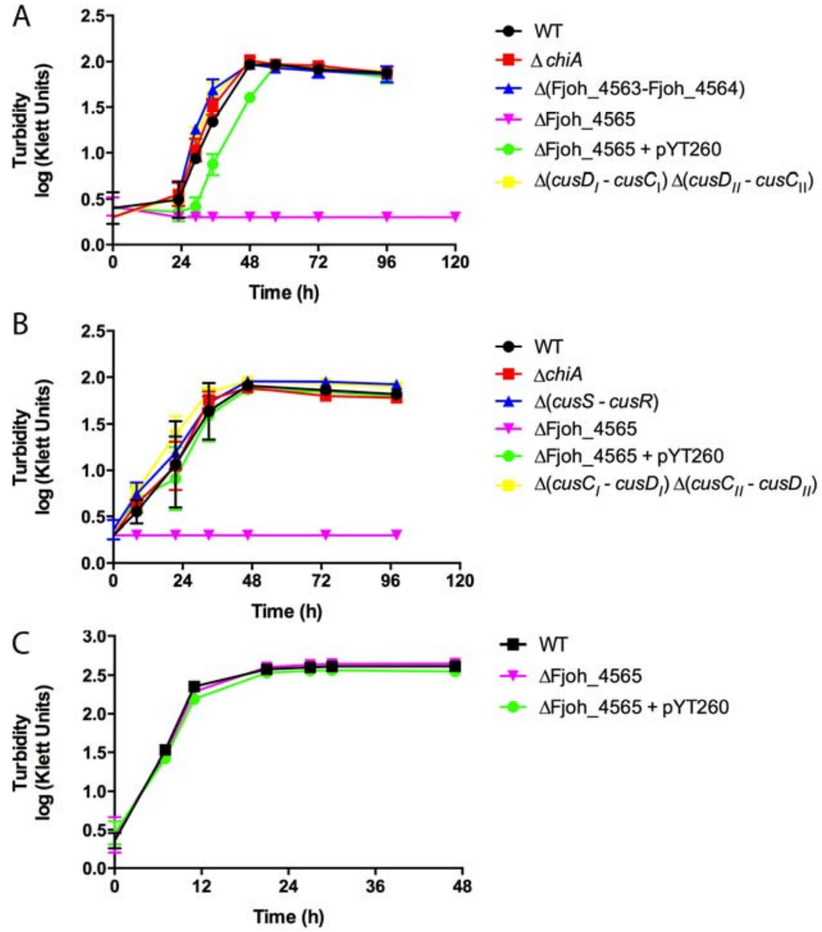

Figure S4: Growth curves of wild type cells and predicted cytoplasmic inner membrane sugar transporter mutants. pYT260 expresses the predicted cytoplasmic membrane sugar transporter Fjoh_4565. Cells (0.1 ml, OD_{600}=1.0) were introduced into 50 ml of medium in 250-ml side-arm flasks and incubated with shaking at 25°C. A: Stanier medium supplemented with 0.1% glucose as sole source of carbon and energy. B: Stanier medium supplemented with 0.1% N-acetylglucosamine as sole source of carbon and energy. C: CYE medium. Growth presented as log (Klett Units). Growth curves were performed in triplicate and error bars indicate standard deviations.

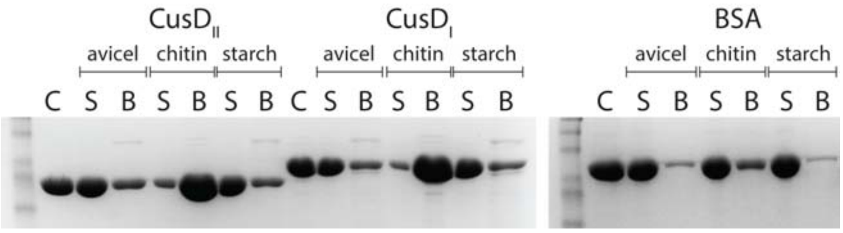

Figure S5: Binding analysis of the CusD proteins to insoluble polysaccharides. Lanes are labelled as C for control (protein incubated in buffer), S for soluble proteins after incubation with polysaccharides, and B for bound proteins, where the polysaccharides had been washed once in buffer and subsequently resuspended in SDS-PAGE loading buffer to release bound proteins. Bovine serum albumin (BSA) was used as a non-binding reference protein.

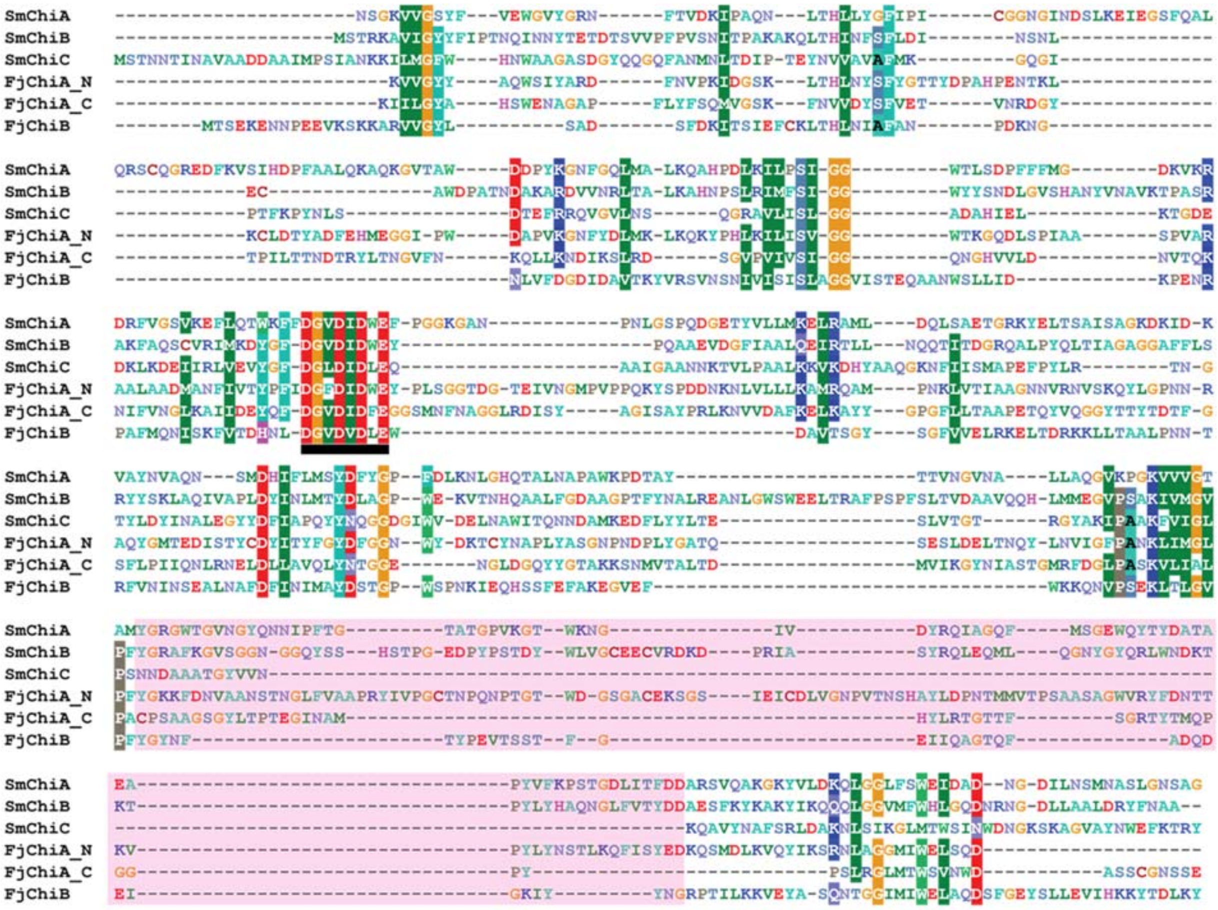

Figure S6: Section of a structural alignment of Serratia marcescens chitinases (ChiA, B and C) to which the ChiUL chitinase domains were added. The red box highlights the α+β-insert region. The characteristic catalytic motif, DXXDXDXE, is indicated by a black bar. The alignment was prepared using Clustal Omega [8], manually edited and displayed using BioEdit.

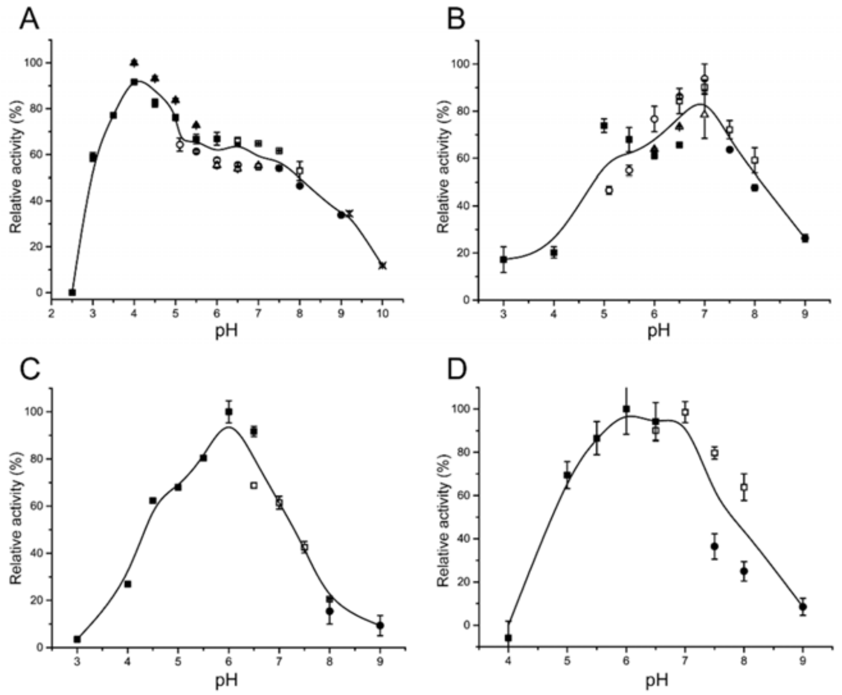

Figure S7: pH profiles for all enzymes. A: ChiA_N, B: ChiA_C, C: ChiB, D: GH20. The buffers (50 mM) used were: citrate (closed squares), sodium phosphate (open squares), tris (closed circles), MES (open circles), sodium acetate (closed triangles), Bis-Tris (open triangles), and sodium carbonate (crosses). The substrate was chitotetraose and the incubation temperature was 25°C. Data points are the average of duplicate experiments and error bars represent the standard error of the mean.

References

1. Bolivar F, Backman K: **Plasmids of *Escherichia coli* as cloning vectors.** *Methods Enzymol* 1979, **68:**245-267.
2. Kharade SS, McBride MJ: ***Flavobacterium johnsoniae* chitinase ChiA is required for chitin utilization and is secreted by the type IX secretion system.** *J Bacteriol* 2014, **196:**961-970.
3. Rhodes RG, Pucker HG, McBride MJ: **Development and use of a gene deletion strategy for *Flavobacterium johnsoniae* to identify the redundant gliding motility genes *remF*, *remG*, *remH*, and *remI*.** *J Bacteriol* 2011, **193:**2418-2428.
4. Agarwal S, Hunnicutt DW, McBride MJ: **Cloning and characterization of the *Flavobacterium johnsoniae* (*Cytophaga johnsonae*) gliding motility gene, *gldA*.** *Proc Natl Acad Sci USA* 1997, **94:**12139-12144.
5. Figurski DH, Helinski DR: **Replication of an origin-containing derivative of plasmid RK2 dependent on a plasmid function provided in trans.** *Proc Natl Acad Sci U S A* 1979, **76:**1648-1652.
6. Suzuki K, Sugawara N, Suzuki M, Uchiyama T, Katouno F, Nikaidou N, Watanabe T: **Chitinases A, B, and C1 of *Serratia marcescens* 2170 produced by recombinant *Escherichia coli*: enzymatic properties and synergism on chitin degradation.** *Biosci Biotechnol Biochem* 2002, **66:**1075-1083.
7. Suzuki K, Taiyoji M, Sugawara N, Nikaidou N, Henrissat B, Watanabe T: **The third chitinase gene (*chiC*) of *Serratia marcescens* 2170 and the relationship of its product to other bacterial chitinases.** *Biochem J* 1999, **343:**587-596.
8. Sievers F, Wilm A, Dineen D, Gibson TJ, Karplus K, Li W, Lopez R, McWilliam H, Remmert M, Soding J, et al: **Fast, scalable generation of high-quality protein multiple sequence alignments using Clustal Omega.** *Mol Syst Biol* 2011, **7:**539.
